# Cerebrospinal fluid biomarkers for assessing Huntington disease onset and severity

**DOI:** 10.1093/braincomms/fcac309

**Published:** 2022-11-25

**Authors:** Nicholas S Caron, Arsalan S Haqqani, Akshdeep Sandhu, Amirah E Aly, Hailey Findlay Black, Jeffrey N Bone, Jodi L McBride, Abedelnasser Abulrob, Danica Stanimirovic, Blair R Leavitt, Michael R Hayden

**Affiliations:** Centre for Molecular Medicine and Therapeutics, BC Children’s Hospital Research Institute, Department of Medical Genetics, University of British Columbia, Vancouver, BC V5Z 4H4, Canada; Human Health Therapeutics Research Centre, National Research Council of Canada, Ottawa, ON K1A 0R6, Canada; BC Children’s Hospital Research Institute, University of British Columbia, Vancouver, BC V5Z 4H4, Canada; Centre for Molecular Medicine and Therapeutics, BC Children’s Hospital Research Institute, Department of Medical Genetics, University of British Columbia, Vancouver, BC V5Z 4H4, Canada; Centre for Molecular Medicine and Therapeutics, BC Children’s Hospital Research Institute, Department of Medical Genetics, University of British Columbia, Vancouver, BC V5Z 4H4, Canada; BC Children’s Hospital Research Institute, University of British Columbia, Vancouver, BC V5Z 4H4, Canada; Division of Neuroscience, Oregon National Primate Research Center, Beaverton, OR 97006, USA; Department of Behavioral Neuroscience, Oregon Health and Science University, Portland, OR 97239, USA; Human Health Therapeutics Research Centre, National Research Council of Canada, Ottawa, ON K1A 0R6, Canada; Human Health Therapeutics Research Centre, National Research Council of Canada, Ottawa, ON K1A 0R6, Canada; Centre for Molecular Medicine and Therapeutics, BC Children’s Hospital Research Institute, Department of Medical Genetics, University of British Columbia, Vancouver, BC V5Z 4H4, Canada; Centre for Molecular Medicine and Therapeutics, BC Children’s Hospital Research Institute, Department of Medical Genetics, University of British Columbia, Vancouver, BC V5Z 4H4, Canada

**Keywords:** Huntington disease, biomarkers, CSF, neurofilament light, proenkephalin

## Abstract

The identification of molecular biomarkers in CSF from individuals affected by Huntington disease may help improve predictions of disease onset, better define disease progression and could facilitate the evaluation of potential therapies. The primary objective of our study was to investigate novel CSF protein candidates and replicate previously reported protein biomarker changes in CSF from Huntington disease mutation carriers and healthy controls. Our secondary objective was to compare the discriminatory potential of individual protein analytes and combinations of CSF protein markers for stratifying individuals based on the severity of Huntington disease. We conducted a hypothesis-driven analysis of 26 pre-specified protein analytes in CSF from 16 manifest Huntington disease subjects, eight premanifest Huntington disease mutation carriers and eight healthy control individuals using parallel-reaction monitoring mass spectrometry. In addition to reproducing reported changes in previously investigated CSF biomarkers (NEFL, PDYN, and PENK), we also identified novel exploratory CSF proteins (C1QB, CNR1, GNAL, IDO1, IGF2, and PPP1R1B) whose levels were altered in Huntington disease mutation carriers and/or across stages of disease. Moreover, we report strong associations of select CSF proteins with clinical measures of disease severity in manifest Huntington disease subjects (C1QB, CNR1, NEFL, PDYN, PPP1R1B, and TTR) and with years to predicted disease onset in premanifest Huntington disease mutation carriers (ALB, C4B, CTSD, IGHG1, and TTR). Using receiver operating characteristic curve analysis, we identified PENK as being the most discriminant CSF protein for stratifying Huntington disease mutation carriers from controls. We also identified exploratory multi-marker CSF protein panels that improved discrimination of premanifest Huntington disease mutation carriers from controls (PENK, ALB and NEFL), early/mid-stage Huntington disease from premanifest mutation carriers (PPP1R1B, TTR, CHI3L1, and CTSD), and late-stage from early/mid-stage Huntington disease (CNR1, PPP1R1B, BDNF, APOE, and IGHG1) compared with individual CSF proteins. In this study, we demonstrate that combinations of CSF proteins can outperform individual markers for stratifying individuals based on Huntington disease mutation status and disease severity. Moreover, we define exploratory multi-marker CSF protein panels that, if validated, may be used to improve the accuracy of disease-onset predictions, complement existing clinical and imaging biomarkers for monitoring the severity of Huntington disease, and potentially for assessing therapeutic response in clinical trials. Additional studies with CSF collected from larger cohorts of Huntington disease mutation carriers are needed to replicate these exploratory findings.

## Teaser

Caron *et al*. report novel multi-marker CSF protein combinations that accurately discriminate between individuals based on Huntington’s disease mutation status and disease severity. These exploratory CSF biomarker panels may improve the accuracy of disease-onset predictions and complement clinical and imaging biomarkers for monitoring disease severity and therapeutic response.

## Graphical Abstract

**Graphical abstract:**
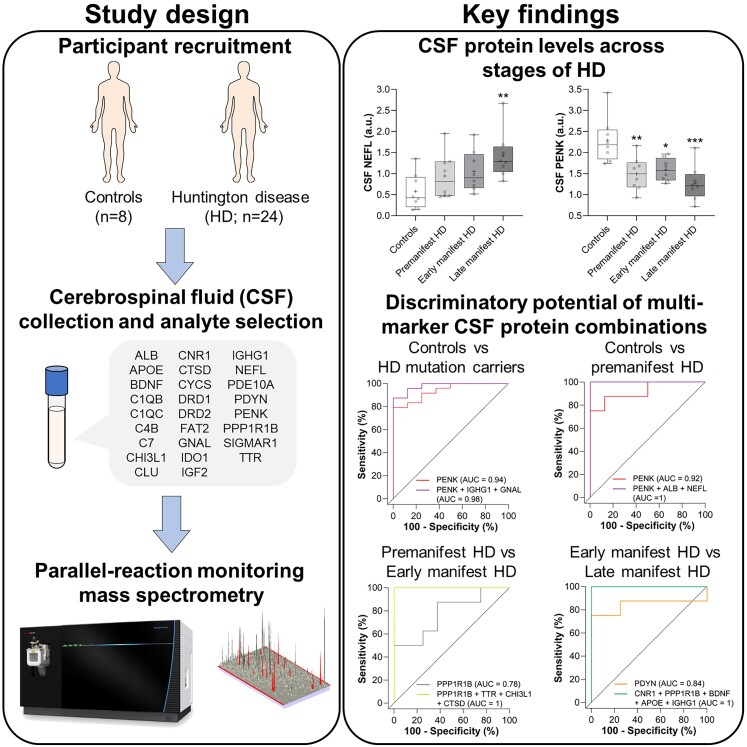

## Introduction

Huntington disease (HD) is an autosomal dominant neurodegenerative disease caused by a cytosine–adenine–guanine (CAG) expansion in the *HTT* gene that codes for an abnormal polyglutamine tract in the huntingtin protein (HTT).^1^ Polyglutamine-expanded mutant huntingtin (mHTT), the primary pathogenic cause of HD, leads to the progressive loss of neuronal populations in the striatum, as well as other structures of the basal ganglia and the cerebral cortex.^2–7^

HD typically manifests in the clinic as an adult-onset disease with affected individuals presenting with cognitive, motor and psychiatric disturbances.^8^ Prior to clinical diagnosis, there is a premanifest or prodromal stage of HD when cellular dysfunction and progressive neurodegeneration are occurring in the brain but no overt symptoms are present. Age-of-onset, a time point when HD mutation carriers develop unequivocal motor signs of HD, is inversely correlated with CAG repeat length in expanded *HTT*,^9^ enabling broad predictions of disease onset.^10^ However, CAG repeat length only accounts for 50–60% of the variability,^10,11^ with other genetic and environmental factors reported to modify age-of-onset.^12–15^

To date, there are no approved therapies to delay the onset or slow the progression of HD. Therapeutic approaches targeting the cause of HD, the CAG expanded *HTT* gene and its products, or downstream processes associated with the pathogenesis of HD, are currently in clinical development. Such therapies may be most effective if intervention is initiated prior to clinical onset and significant neurodegeneration in the brain.

CSF is an accessible biofluid whose molecular composition reflects structural and functional changes in the brain, making it a promising biofluid for biomarker discovery in HD and other neurodegenerative disorders. In HD, CSF biomarkers may offer the potential to monitor cell-type and/or pathway-specific pathophysiological alterations in the CNS over the natural history of the disease. Sensitive CSF biomarkers that reflect early cellular dysfunction or neurodegeneration in the brain during the premanifest stage of HD may help improve the accuracy of disease-onset predictions and could potentially guide the appropriate timing for therapeutic intervention. Moreover, such biomarkers could be used to complement existing clinical^16,17^ and imaging-based^18,19^ biomarkers for monitoring disease progression and assessing the efficacy of candidate therapies in HD clinical trials.

Several promising molecular biomarkers have been identified in CSF and/or blood that is altered in HD (reviewed in^20,21^). However, only mHTT^22–26^ and NEFL^24,25,27–32^ have been used in HD clinical trials.

CSF mHTT increases with disease progression^25^ and its levels correlate with clinical measures of disease severity.^22–25^ Importantly, a dose-dependent reduction of CSF mHTT was observed in a Phase I/IIa clinical trial evaluating a *HTT*-targeted antisense oligonucleotide (tominersen) delivered by intrathecal infusion, suggesting that CSF mHTT could be a valuable biomarker to assess target engagement in the CNS.^33^ However, preliminary findings from the halted Phase III trial evaluating the efficacy of tominersen (NCT03761849) suggest that a reduction of CSF mHTT alone may not predict clinical benefit.

NEFL in biofluids is a biomarker of neuronal injury, with elevated NEFL levels in CSF and blood reported in HD,^24,25,27–31^ as well as other neurological diseases (reviewed in^34^). In HD, NEFL levels in biofluids are correlated with clinical and imaging measures of disease^24,27^ and are a strong prognostic biomarker of disease onset, progression and brain atrophy in HD patients.^25,27,29^ Notably, NEFL is being used in HD clinical trials as an exploratory biomarker to monitor disease progression and to assess therapeutic efficacy. However, it remains unknown if NEFL in biofluids will respond to candidate therapies in a manner that reflects clinical benefit.

We conducted a hypothesis-driven analysis of 26 pre-specified proteins in the CSF from 16 manifest HD (manHD) patients, 8 premanifest HD (preHD) and 8 control individuals using nanoflow liquid chromatography-coupled parallel-reaction monitoring mass spectrometry (nanoLC-PRM-MS). This methodology allows for the simultaneous identification and quantification of more than 30 peptides at attomole concentrations within a single run,^35–37^ allowing for reliable monitoring of CSF proteins with high specificity and sensitivity. An initial list of protein candidates was prioritized based on existing literature demonstrating altered levels in the CSF of HD mutation carriers, including C1QC,^38^ C4B,^38^ CHI3L1 (also known as YKL-40),^30,38,39^ CLU,^40,41^ CTSD,^38^ FAT2,^41^ NEFL, PDYN,^42^ PENK,^41^ and TTR.^38,41,43^ Additional protein candidates were selected that, to our knowledge, have not been previously measured in HD CSF but were either reported to have altered expression in the striatum of HD patients,^44–46^ animal models of HD,^47,48^ or have been implicated in the pathogenesis of HD.^49,50^

In this study, we sought to investigate novel CSF protein candidates and replicate previously reported molecular biomarker changes in CSF from HD mutation carriers and healthy controls. We assess potential associations between levels of CSF protein analytes, as well as correlations of candidate CSF proteins with clinical measures of disease severity. Finally, we compare the discriminatory potential of individual CSF proteins and combinations of CSF protein markers to assess their sensitivity and specificity for stratifying subjects based on HD mutation status and disease severity.

## Materials and methods

### Study participants

A hypothesis-driven analysis of protein analytes was performed in CSF from 16 manHD, eight preHD and eight healthy control individuals recruited through the University of British Columbia’s Centre for Huntington Disease. PreHD was defined as individuals with *HTT* CAG repeat expansions >36 and a Unified Huntington’s Disease Rating Scale (UHDRS) diagnostic confidence level (DCL) < 3, whereas manHD was defined as individuals with a *HTT* CAG repeat expansion >36 and a DCL = 4. HD mutation carriers refer to both preHD and manHD individuals. Healthy control individuals with no neurological abnormalities and *HTT* CAG repeat lengths <36 were selected to span the range of ages of HD mutation carriers.

Clinical outcomes including total functional capacity (TFC), total motor score (TMS), verbal fluency (VF), symbol digit modality test (SDMT), and Stroop word reading (SWR) were scored by a trained neurologist using the UHDRS.^16^ CAG-age product (CAP) score or disease burden score was calculated using the formula: (CAG length—35.5) × age.^51^ Predicted age-of-onset estimates in preHD individuals were calculated according to the formula: 21.54 + EXP(9.556—0.146 CAG) and years to predicted disease onset was estimated by subtracting the individual’s age at the time of CSF collection.^10^

### CSF collection

CSF samples from Huntington disease mutation carriers and control individuals were collected at the University of British Columbia’s Centre for Huntington Disease. CSF was obtained by lumbar puncture, examined qualitatively by microscopy and centrifuged to remove cells. The acellular supernatant was aliquoted and frozen at −80°C.

### Study approval and patient consent

All CSF samples were collected under an approved protocol (H14-03131) in accordance with the guidelines of the institutional review board of the University of British Columbia and with the full informed consent of the subjects.

### Parallel-reaction monitoring mass spectrometry

A panel of 26 proteins was measured in CSF by nanoLC-PRM-MS. For sample preparation, each CSF sample was reduced, alkylated, and trypsin digested as previously described^52,53^ and cleaned using detergent removal spin columns (Thermo Fisher Scientific, catalog # 87777) as per the manufacturer’s protocol. The samples were acidified with 1% formic acid (EMD Millipore) and loaded on a reversed-phase UltiMate™ 3000 RSLC-nano System with ProFlow Meter (Thermo Fisher) coupled with Orbitrap Eclipse™ Tribrid™ mass spectrometer (Thermo Fisher) for analysis with a nano-electrospray interface operated in positive ion mode. Prior to PRM analysis, 112 peptides corresponding to 2–15 peptides per protein (Supplementary Table 1) were identified and validated using data-dependent acquisition (DDA) and split among four nanoLC-PRM-MS runs. The DDA and nanoLC-PRM-MS analysis involved injection and loading of ∼0.1–0.2 μg of the peptide sample onto a 300 µm I.D. × 0.5 mm 3 µm PepMaps® C18 trap (Thermo Fisher) followed by separation on a 100 µm I.D. × 10 cm 1.7 µm BEH130C18 nanoLC column (Waters, Milford, MA, USA). The eluted peptides were ionized by electrospray ionization for either DDA or nanoLC-PRM-MS analysis and the data for MS/MS was acquired in the Orbitrap on ions with mass-to-charge values between 375 and 1800 at a resolution of 60 000 followed by higher-energy collisional dissociation fragmentation and PRM scans. Raw data extraction and data analysis were performed using Skyline software version 3.7 (https://skyline.ms) and MatchRx software version 3.0 as previously described.^53^ The extracted peptide intensities (peak areas) were normalized against a median intensity value calculated from all peptide intensities in each run.

### Statistical analysis

Statistical analyses were performed using GraphPad Prism 9 (GraphPad) and R statistical software,^54^ using the Caret^55^ and MixOmics^56^ packages for modelling. Alpha values of <0.05 were considered significant for all analyses.

Comparisons of demographic characteristics and clinical measures between groups were assessed by ANOVA and Fisher’s least significant difference test. Mean values ± standard deviation (SD) for each group are presented. CAP scores were compared between preHD and manHD individuals using a two-tailed *t*-test. Differences in gender distributions between groups were assessed using Pearson’s *χ*^2^ test.

Age, sex and CAG repeat length were considered potential confounding factors for comparisons of CSF protein levels between groups. The relationship of normalized CSF protein concentrations with age and sex was evaluated in control individuals using either Pearson’s correlation or independent unpaired *t*-tests, respectively. The association of CSF protein levels with CAG repeat length in all HD mutation carriers was assessed using Pearson’s correlation. Only age was found to be significantly associated with CSF protein levels and was included as a covariate for all subsequent analyses. Normalized CSF protein concentrations for all individuals were adjusted for age using linear regression.

Pre-specified analyses comparing age-adjusted CSF protein levels between controls and all HD mutation carriers were performed using general linear models (GLMs) bootstrapped with 1000 repetitions. *P*-values and the percentage of events in 1000 bootstrap repetitions that the variable was selected with *P* < 0.05 are reported for each comparison. Odds ratios (OR) and 95% confidence intervals (CIs) for statistically significant comparisons are presented.

Comparisons across disease stages were performed by analysis of covariance (ANCOVA) including age as a covariate, and *F* statistics, degrees of freedom, and *P*-values for each comparison are reported. *Post hoc* tests between disease stages were performed using Tukey’s test to correct for multiple comparisons and mean difference (MD) effect sizes, 95% CI and *P*-values for statistically significant comparisons are reported.

Associations of clinical measures of disease severity with CSF protein levels were assessed in manHD individuals using Spearman’s partial rank correlation including age as a covariate, with the exception of CAP score which was evaluated in all HD mutation carriers using unadjusted data. The relationship of CSF protein levels with years to predicted disease onset was assessed in preHD subjects using Pearson’s correlation on unadjusted data. Associations between each of the 26 CSF protein analytes were evaluated in all HD mutation carriers using Pearson’s partial correlation including age as a covariate. Coefficient values (Spearman’s *ρ* or Pearson’s *r*) from ±0.50 to ±1 were considered strong correlations, ± 0.30 to ±0.49 were considered moderate correlations and ±0.10 to ±0.29 were considered weak correlations. *P*-values <0.05 define correlations significantly different than 0.

The sensitivity (% of individuals with the target condition that the test correctly identifies as positive) and specificity (% of individuals without the target condition that the test correctly identifies as negative) of each individual CSF protein for discriminating between disease groups/stages were assessed using receiver operating characteristic (ROC) curve analysis, and the corresponding area under the curve (AUC) values were computed as a measure of discriminatory performance or accuracy. CSF proteins with AUC = 0.8–1 were considered as being classifiers with high discriminatory ability, values of 0.7–0.8 as having moderate discriminatory ability, and 0.6–0.7 as classifiers with weak discriminatory ability. AUC values, AUC 95% CIs and *P*-values for each test are reported. AUC 95% CI was computed using the Wilson/Brown hybrid method and AUCs were compared as described by DeLong *et al*.^57^

Sparse partial least squares discriminant analysis (sPLS-DA) is a supervised machine learning method that examines the discriminative capacity of multi-dimensional data while selecting features best able to classify samples. For each comparison, the sPLS-DA model was tuned to find the appropriate number of components and variables using 50 × 3-fold repeated cross-validation. Then, a final sPLS-DA model was fit using the optimal number of proteins for the respective optimal number of components, as determined during the tuning phase to avoid overfitting. This entire process was bootstrapped with 1000 repetitions to assess the variability and stability of the final models. ROC curves for the final sPLS-DA model were then generated and AUC values, AUC 95% CI and *P*-values are reported.

Multi-marker ROC curves were generated using the CombiROC analytical tool.^58^ Data sets comprising age-adjusted values from up to 10 CSF proteins were uploaded into the web-based interface, and analysis was performed without further processing of the data. Test-signal cut-offs, as well as sensitivity and selectivity thresholds, were adjusted for different group comparisons. ROC curves with combinations of up to five proteins were plotted and AUC values are reported.

## Results

### Demographics and clinical characteristics of study participants

Study participant demographics and clinical scores are summarized in Table 1. Our study included 8 healthy controls, 8 preHD mutation carriers and 16 manHD subjects. A significant age difference between groups was observed, with manHD patients being significantly older than preHD individuals (mean age ± SD = 52.12 ± 11.94 versus 37.18 ± 9.08. *P* = 0.011). Healthy controls were selected to span the age range of HD mutation carriers and no significant age differences were observed compared with either preHD (*P* = 0.119) or manHD subjects (*P* = 0.398). There were no significant differences in sex distributions between groups (*χ*^2^: 0.254, *P* = 0.881) or CAG repeat lengths between preHD and manHD patients (mean CAG ± SD = 43.64 ± 1.51 versus 44.50 ± 2.78. *P* = 0.196).

**Table 1:** Demographics and clinical characteristics of study participants

	Controls *n* = 8	PreHD *n* = 8	ManHD *n* = 16	ANOVA	Controls versus preHD	Controls versus manHD	PreHD versus manHD
	Mean ± SD			*P*-value*			
Age	47.63 ± 14.85	37.88 ± 9.08	52.13 ± 11.94	0.037	0.119	0.398	0.011
Sex (M/F)	4/4	5/3	9/7	N/A	N/A	N/A	N/A
CAG	17.88 ± 1.13	43.64 ± 1.51	44.50 ± 2.78	**<0**.**0001**	**<0**.**0001**	**<0**.**0001**	0.196
BMI	27.06 ± 3.18	29.46 ± 7.81	26.20 ± 6.15	0.477	0.436	0.747	0.229
DCL (*n*)	0 (7), 1 (1)	0 (2), 1 (5), 2 (1)	4 (16)	N/A	N/A	N/A	N/A
CAP	N/A	302.60 ± 65.65	492.80 ± 108.90	N/A	N/A	N/A	**0**.**0002**
TFC	13 ± 0	12.75 ± 0.46	5.25 ± 4.51	**<0**.**0001**	0.879	**<0**.**0001**	**<0**.**0001**
TMS	0.38 ± 1.06	3.25 ± 2.96	57.81 ± 25.49	**<0**.**0001**	0.757	**<0**.**0001**	**<0**.**0001**
VF	42.63 ± 9.13	43.63 ± 9.01	19.50 ± 17.40	**0**.**0002**	0.888	**0**.**0007**	**0**.**0004**
SDMT	51.25 ± 6.80	44.00 ± 9.35	18.56 ± 9.98	**<0**.**0001**	0.124	**<0**.**0001**	**<0**.**0001**
SWR	96.38 ± 6.84	83.50 ± 15.48	53.81 ± 15.76	**<0**.**0001**	0.077	**<0**.**0001**	**<0**.**0001**

Comparisons with *P*-values <0.05 are shown in bold. BMI = body mass index; CAG = cytosine–adenine–guanine; CAP = CAG-age product; DCL = diagnostic confidence level; manHD = manifest Huntington disease; preHD = premanifest Huntington disease; SD = standard deviation; SDMT = symbol digit modality test; SWR = Stroop word reading; TFC = total functional capacity; TMS = total motor score; VF = verbal fluency. *P-values presented are not corrected for multiple comparisons.

### Comparison of CSF protein levels across disease stages

We pre-specified 26 protein analytes to measure in CSF from controls and HD mutation carriers, including previously investigated CSF proteins, as well as exploratory candidate proteins that, to our knowledge, have never been investigated in HD CSF (Table 2).

**Table 2:** Protein analytes measured in CSF from HD mutation carriers and control individuals

CSF protein	Biological function(s)	Brain-enriched expression	Fold change^a^ (HD mutation carriers / control)	*P*-value (% selected)^b^
NEFL	Cytoskeleton/axonal transport	Yes	1.97	**0.031 (60.2%)**
GNAL^c^	Signal transduction	Yes	1.52	**0.043 (51.7%)**
DRD1^c^	Synaptic transmission/neuron growth	Yes	1.42	0.062 (44.5%)
IGF2^c^	Carbohydrate metabolism/growth factor	No	1.36	**0.024 (67.4%)**
IGHG1	Immune response	No	1.28	**0.015 (77.6%)**
CHI3L1	Immune response	No	1.25	0.371 (2.5%)
C7^c^	Complement system/immune response	No	1.24	0.111 (28.5%)
FAT2	Cell adhesion/migration	Yes	1.14	0.213 (24.3%)
ALB	Transport protein	No	1.14	0.068 (42.1%)
PDE10A^c^	Signal transduction	Yes	1.07	0.611 (4.8%)
CLU	Apoptosis/oxidative stress/immune response	No	1.05	0.723 (1.1%)
C4B	Complement system/immune response	No	1.01	0.953 (3.3%)
CYCS^c^	Energy metabolism/apoptosis	No	0.99	0.961 (1.8%)
DRD2^c^	Synaptic transmission/axonogenesis/neuron migration	Yes	0.99	0.947 (4.6%)
SIGMAR1^c^	Lipid transport/G-protein signalling/apoptosis	No	0.94	0.620 (2.3%)
TTR	Signal transduction/transport protein	No	0.90	0.314 (11.4%)
C1QC	Complement system/immune response	No	0.89	0.583 (4.1%)
IDO1^c^	Apoptosis/immune response	No	0.89	0.156 (22.8%)
CNR1^c^	Apoptosis/synaptic transmission/immune response	Yes	0.89	0.435 (2.6%)
CTSD	Protein degradation/apoptosis/immune response	No	0.87	**0.044 (52.3%)**
C1QB^c^	Complement system/immune response	No	0.86	0.222 (14.3%)
PPP1R1B^c^	Signal transduction	Yes	0.82	0.204 (19.8%)
APOE^c^	Lipid transport/synapse organization	No	0.80	0.139 (24.8%)
BDNF	Synapse assembly/axon guidance/neuronal health	Yes	0.78	0.056 (45.9%)
PDYN	Neuropeptide signalling	Yes	0.76	**0.018 (65.6%)**
PENK	Neuropeptide signalling	No	0.63	**0.011 (84.9%)**

Comparisons with *P*-values <0.05 are shown in bold. ^a^Fold changes represent the ratio of age-adjusted means between HD mutation carriers and controls. ^b^Represents the percentage of events in 1000 bootstrap repetitions that the variable was selected with a *P*-value <0.05. ^c^Exploratory CSF markers not previously investigated in CSF from HD mutation carriers. HD = Huntington disease

We utilized a nanoLC-PRM-MS method to quantify unique peptides derived from each of the 26 CSF proteins with high sensitivity and specificity. For each protein, 2–15 unique peptides were measured in parallel. A complete list of peptide sequences measured by nanoLC-PRM-MS is presented in Supplementary Table 1. We observed moderate to strong positive correlations between normalized unadjusted values for each peptide from all protein candidates assessed, suggesting a reliable measurement of these proteins in CSF (Supplementary Table 1). Mean normalized peptide concentrations for each CSF protein were then adjusted to control for the effects of age, and residuals were used for subsequent analyses.

We first compared age-adjusted values of CSF proteins in all HD mutation carriers (includes preHD and manHD individuals) and controls using bootstrapped GLMs (Table 2). We found that NEFL (OR = −2.785, 95% CI: −5.821 to −0.650, *P* = 0.031), GNAL (OR = −3.134, 95% CI: −6.748 to −0.512, *P* = 0.043), IGF2 (OR = −7.194, 95% CI: −14.816 to −1.798, *P* = 0.024), and IGHG1 (OR = −8.026, 95% CI: −15.954 to −2.556, *P* = 0.015) were significantly increased, whereas CTSD (OR = 4.855, 95% CI: 0.821–10.688, *P* = 0.044), PDYN (OR = 3.912, 95% CI: 1.209–7.821, *P* = 0.018), and PENK (OR = 5.673, 95% CI: 2.301–11.464, *P* = 0.011) were significantly decreased in CSF from HD mutation carriers compared with controls. We also observed trends towards increased levels DRD1 (OR = −3.719, 95% CI: −8.216 to −0.194, *P* = 0.062) and ALB (OR = −5.534, 95% CI: −12.505 to −0.258, *P* = 0.068), and decreased levels of BDNF (OR = 2.649, 95% CI: 0.13–5.716, *P* = 0.056) in HD mutation carriers but these did not reach statistical significance.

**Figure 1:**
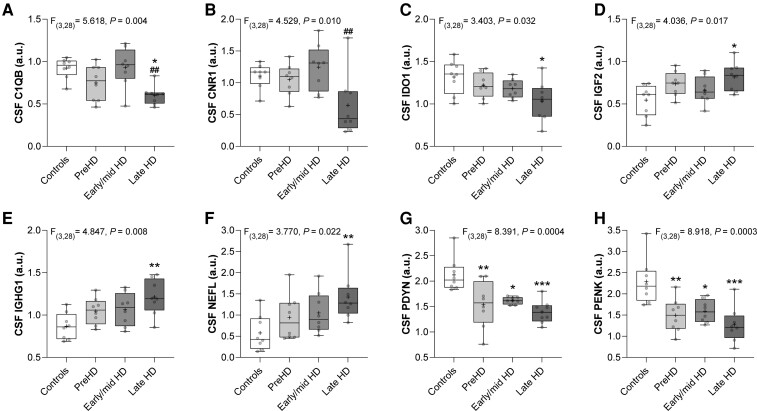
**Comparison of CSF protein levels across disease stages**. Box and whisker plots comparing normalized CSF protein levels between controls (*n* = 8), preHD (*n* = 8), early/mid HD (TFC >5; *n* = 8), and late HD (TFC <5; *n* = 8) individuals. Intergroup differences were assessed using ANCOVA including age as a covariate and summary statistics are shown at the top of each plot. *Post hoc* tests were performed using Tukey’s test to correct for multiple comparisons. (**A**) C1QB (*P = 0.010 compared with controls, ^##^*P* = 0.008 compared with early/mid HD), (**B**) CNR1 (^##^*P* = 0.008 compared with early/mid HD), (**C**) IDO1 (*P = 0.020 compared with controls), (**D**) IGF2 (*P = 0.012 compared with controls), (**E**) IGHG1 (**P = 0.004 compared with controls), (**F**) NEFL (**P = 0.002 compared with controls), (**G**) PDYN (*P = 0.012, **P = 0.004, ***P = 0.0003 compared with controls) and (**H**) PENK (*P = 0.012, **P = 0.004, ***P = 0.0002 compared with controls). Individual data points are plotted for each group. Boxes show 25th to 75th percentiles, the central band denotes the median, the plus sign denotes the mean, and the whiskers show the minimum and maximum values. ANCOVA = analysis of covariance; a.u. = arbitrary units; preHD = premanifest Huntington disease.

We next investigated whether CSF protein levels were altered across stages of the disease. HD mutation carriers were divided based on DCL into preHD (DCL < 3) and manHD groups (DCL = 4), and the manHD group was further stratified based on TFC score into early/mid HD (TFC > 5) and late HD (TFC < 5) groups. A comparison of age-adjusted CSF protein levels was performed between controls, preHD, early/mid HD and late HD groups by ANCOVA followed by *post hoc* analysis using Tukey’s test to correct for multiple comparisons (Supplementary Table 2). We identified eight CSF proteins that were significantly altered across disease stages (Fig. 1).

Levels of C1QB were significantly decreased in late HD compared with controls (MD = 0.322, 95% CI: 0.064–0.580, *P = 0.010), and late HD compared with early/mid HD (Fig. 1A, MD = 0.331, 95% CI: 0.073–0.589, ^##^*P* = 0.008). CNR1 levels were significantly reduced in late HD compared with early/mid HD (Fig. 1B, MD = 0.601, 95% CI: 0.133–1.070, ^##^*P* = 0.008) and a strong trend towards a reduction in late HD compared with controls was observed but did not reach *post hoc* significance (MD = 0.461, 95% CI: −0.007–0.930, *P* = 0.055).

IDO1 levels were significantly decreased (Fig. 1C, MD = 0.285, 95% CI: 0.036–0.533, *P = 0.020), whereas IGF2 (Fig. 1D, MD = −0.274, 95% CI: −0.497 to −0.050, *P = 0.012), IGHG1 (Fig. 1E, MD = −0.344, 95% CI: −0.592 to −0.097, **P = 0.004) and NEFL (Fig. 1F, MD = −0.848, 95% CI: −1.368 to −0.327, **P = 0.002) were significantly increased in late HD compared with control individuals. Trends towards increased NEFL in early/mid HD compared with controls (MD = −0.473, 95% CI: −0.994–0.048, *P* = 0.073) and late HD compared with preHD (MD = −0.483, 95% CI: −1.003–0.038, *P* = 0.068) were observed but did not reach *post hoc* significance.

Levels of PDYN (Fig. 1G) and PENK (Fig. 1H) were significantly decreased in preHD (PDYN: MD = 0.614, 95% CI: 0.176–1.052, **P = 0.004, PENK: MD = 0.799, 95% CI: 0.229–1.369, **P = 0.004), early/mid HD (PDYN: MD = 0.537, 95% CI: 0.099–0.975, *P = 0.012, PENK: MD = 0.698, 95% CI: 0.128–1.268, *P = 0.012), and late HD compared with controls (PDYN: MD = 0.761, 95% CI: 0.323–1.199, ***P = 0.0003, PENK: MD = 1.021, 95% CI: 0.451–1.591, ***P = 0.0002).

PPP1R1B (also known as DARPP-32) levels were significantly altered across disease stages (Supplementary Table 2, *P* = 0.042) and showed a trend towards decreased levels in late HD compared with early/mid HD groups (MD = 0.3682, 95% CI: −0.029–0.765, *P* = 0.076). BDNF levels showed a strong trend towards a reduction in late HD compared with controls, but this difference did not reach statistical significance (MD = 0.433, 95% CI: −0.004–0.870, *P* = 0.053).

### Correlations of CSF protein levels with clinical measures of disease severity

Correlations of CSF protein levels with CAP score, an age-dependent measure of cumulative exposure to CAG expanded *HTT*, were performed on unadjusted values using Spearman’s rank correlation in all HD mutation carriers (Table 3). C4B (*ρ* = 0.44, *P* = 0.031), NEFL (*ρ* = 0.44, *P* = 0.033), IDO1 (*ρ* = −0.45, *P* = 0.029), and PENK (*ρ* = −0.41, *P* = 0.048) showed significant correlations with this measure of disease burden.

**Table 3:** Correlations of CSF protein levels with clinical measures of disease severity

	CAP		TFC		TMS		VF		SDMT		SWR	
	*ρ*	*P*-value	*ρ*	*P*-value	*ρ*	*P*-value	*ρ*	*P*-value	*ρ*	*P*-value	*ρ*	*P*-value
ALB	−0.27	0.196	0.07	0.791	0.15	0.569	0.10	0.703	−0.12	0.665	0.02	0.953
APOE	−0.09	0.661	0.23	0.386	0.08	0.781	0.13	0.641	0.37	0.160	−0.04	0.890
BDNF	−0.08	0.698	0.45	0.083	−0.25	0.356	0.32	0.222	0.48	0.064	0.21	0.422
C1QB	0.00	0.986	0.31	0.236	−0.11	0.678	0.19	0.469	0.51	**0**.**045**	0.09	0.742
C1QC	0.11	0.623	0.00	0.998	0.30	0.252	−0.13	0.637	0.14	0.594	−0.27	0.303
C4B	0.44	**0**.**031**	0.34	0.196	−0.37	0.164	0.10	0.699	0.31	0.236	0.28	0.287
C7	0.22	0.295	−0.09	0.749	0.29	0.279	−0.27	0.308	−0.04	0.881	−0.36	0.175
CHI3L1	0.40	0.054	0.30	0.252	−0.03	0.910	0.03	0.912	0.38	0.142	−0.10	0.720
CLU	−0.01	0.965	0.12	0.645	0.07	0.807	0.07	0.802	0.31	0.236	−0.08	0.767
CNR1	−0.24	0.264	0.58	**0**.**021**	−0.38	0.142	0.52	**0**.**041**	0.56	**0**.**026**	0.28	0.292
CTSD	−0.32	0.126	0.07	0.783	−0.06	0.833	0.25	0.348	0.21	0.440	0.13	0.617
CYCS	0.24	0.258	0.08	0.757	0.21	0.437	−0.09	0.737	0.20	0.447	−0.18	0.492
DRD1	0.10	0.658	0.05	0.857	−0.18	0.495	0.12	0.649	−0.10	0.713	0.11	0.670
DRD2	0.15	0.473	0.27	0.312	−0.42	0.106	0.18	0.494	0.24	0.359	0.25	0.349
FAT2	0.14	0.509	−0.05	0.852	0.22	0.411	−0.14	0.600	−0.01	0.984	−0.21	0.442
GNAL	0.09	0.671	−0.07	0.800	−0.02	0.941	0.05	0.850	−0.21	0.426	0.14	0.598
IDO1	−0.45	**0**.**029**	0.43	0.100	−0.27	0.313	0.50	0.053	0.32	0.224	0.21	0.429
IGF2	0.18	0.387	−0.40	0.122	0.40	0.126	−0.37	0.161	−0.36	0.164	−0.42	0.106
IGHG1	0.07	0.759	−0.28	0.285	0.24	0.365	−0.15	0.589	−0.33	0.205	−0.15	0.570
NEFL	0.44	**0**.**033**	−0.23	0.392	0.32	0.231	−0.40	0.123	−0.22	0.405	−0.50	**0**.**048**
PDE10A	0.08	0.718	0.21	0.424	−0.27	0.313	0.14	0.608	0.10	0.700	0.27	0.300
PDYN	−0.26	0.212	0.53	**0**.**035**	−0.36	0.171	0.55	**0**.**031**	0.57	**0**.**023**	0.43	0.100
PENK	−0.41	**0**.**048**	0.35	0.182	−0.05	0.858	0.31	0.238	0.42	0.111	0.14	0.605
PPP1R1B	0.12	0.563	0.54	**0**.**034**	−0.34	0.200	0.41	0.113	0.53	**0**.**038**	0.23	0.393
SIGMAR1	−0.22	0.303	0.32	0.222	−0.33	0.205	0.36	0.169	0.08	0.758	0.26	0.320
TTR	−0.20	0.360	0.30	0.262	−0.52	**0**.**043**	0.49	0.056	0.40	0.126	0.50	0.051

Correlations with *P*-values <0.05 are shown in bold. CAP = CAG-age product; SDMT = symbol digit modality test; SWR = Stroop word reading; TFC = total functional capacity; TMS = total motor score; VF = verbal fluency.

The relationship of CSF protein levels with clinical measures of disease severity in manHD individuals was evaluated using Spearman’s partial rank correlation including age as a covariate (Table 3). CNR1 (*ρ* = 0.58, *P* = 0.021), PPP1R1B (*ρ* = 0.54, *P* = 0.034), and PDYN (*ρ* = 0.53, *P* = 0.035) were strongly correlated with TFC in manHD individuals, whereas BDNF (*ρ* = 0.45, *P* = 0.083), IDO1 (*ρ* = 0.43, *P* = 0.100) and IGF2 (*ρ* = −0.40, *P* = 0.122) showed moderate correlations. TTR (*ρ* = −0.52, *P* = 0.043) showed a strong significant negative correlation, and DRD2 (*ρ* = −0.42, *P* = 0.106) and IGF2 (*ρ* = 0.40, *P* = 0.126) were moderately correlated with TMS in manHD subjects.

PDYN (*ρ* = 0.55, *P* = 0.031), CNR1 (*ρ* = 0.52, *P* = 0.041) and IDO1 (*ρ* = 0.50, *P* = 0.053) were strongly correlated with VF score, whereas TTR (*ρ* = 0.49, *P* = 0.056) and PPP1R1B (*ρ* = 0.41, *P* = 0.113) showed moderate positive correlations. PDYN (*ρ* = 0.57, *P* = 0.023), CNR1 (*ρ* = 0.56, *P* = 0.026), PPP1R1B (*ρ* = 0.53, *P* = 0.038) and C1QB (*ρ* = 0.51, *P* = 0.045) showed strong significant correlations, whereas BDNF (*ρ* = 0.48, *P* = 0.064), PENK (*ρ* = 0.42, *P* = 0.111) and TTR (*ρ* = 0.40, *P* = 0.126) showed moderate correlations with SDMT in manHD individuals. Finally, NEFL (*ρ* = −0.50, *P* = 0.048) and TTR (*ρ* = 0.50, *P* = 0.051) showed strong correlations with SWR score, whereas PDYN (*ρ* = 0.43, *P* = 0.0997) and IGF2 (*ρ* = −0.42, *P* = 0.1063) were moderately correlated with this clinical measure in manHD individuals.

We also assessed the relationship of unadjusted CSF protein levels with years to predicted disease onset^10^ in preHD mutation carriers (mean years to predicted onset ± SD = 8.41 ± 8.04) using Pearson’s correlation and found that ALB (*r* = 0.75, *P* = 0.031), C4B (*r* = −0.74, *P* = 0.036), CTSD (*r* = 0.66, *P* = 0.08), IGHG1 (*r* = 0.85, *P* = 0.008) and TTR (*r* = 0.86, *P* = 0.006) showed strong associations with these estimates (Supplementary Table 3).

### Correlations between CSF protein analytes in Huntington disease mutation carriers

The relationship between individual CSF protein analytes in HD mutation carriers was assessed using Pearson’s partial correlation (Supplementary Table 4). Functional enrichment analysis was performed using all 26 CSF proteins to identify overlap in biological processes related to the pathophysiology of Huntington disease.^59^ We observed moderate to strong correlations between levels of CSF proteins involved in neuronal function, motor behaviour, cognition and memory, synapse organization and plasticity, apoptosis/cell death, as well as immune and complement pathway activation.

### Discriminatory potential of CSF protein markers for Huntington disease

We next used ROC curve analysis to evaluate the sensitivity and specificity of each CSF protein for discriminating between either HD mutation carriers and controls, preHD and controls, or manHD and preHD. For each test, AUC values were computed as a measure of discriminatory performance for distinguishing individuals based on HD mutation status and disease severity (Supplementary Table 5).

**Figure 2:**
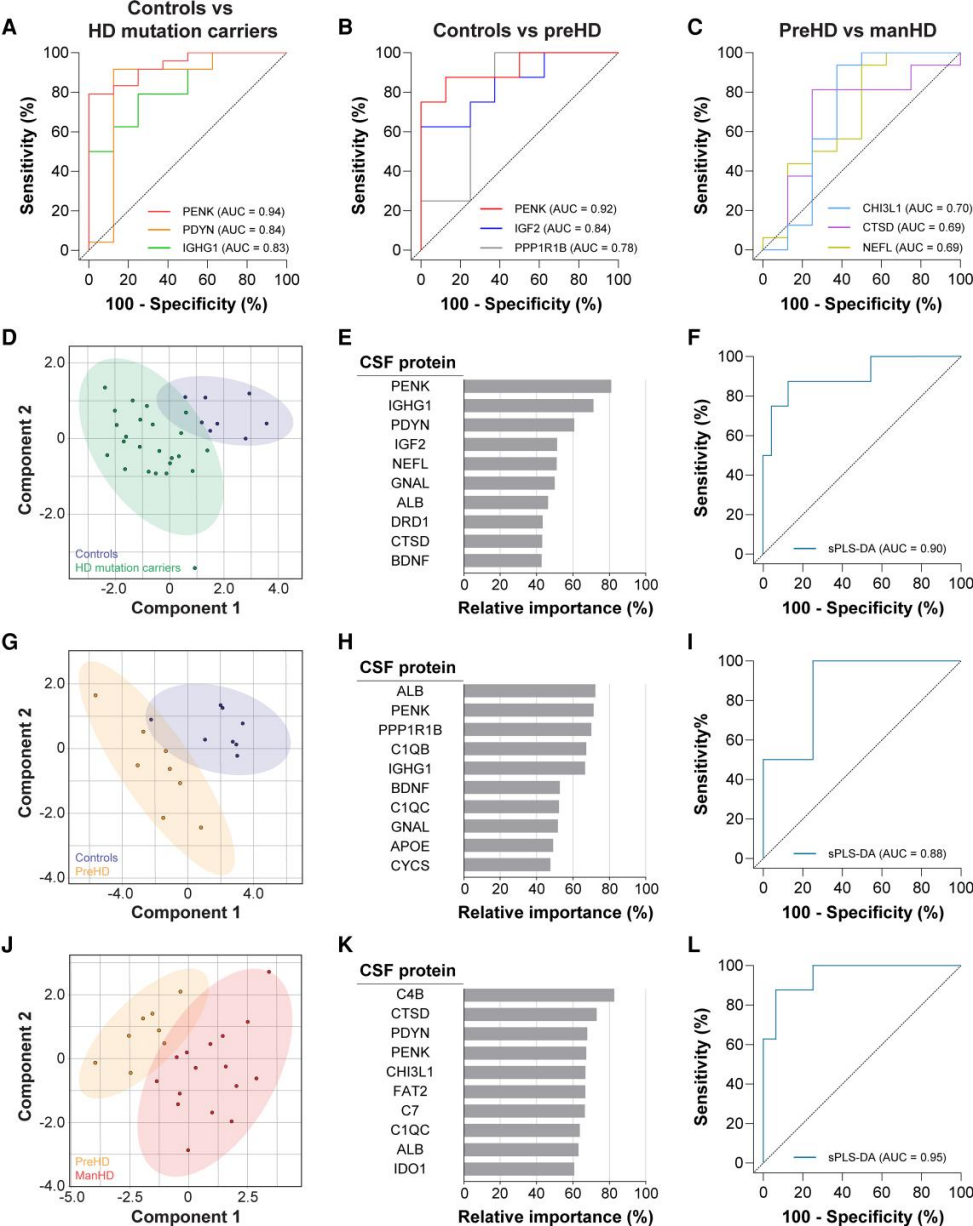
**Discriminatory potential of CSF protein markers for HD.** ROC curves showing the top 3 individual CSF proteins with highest discriminatory power for distinguishing (**A**) HD mutation carriers from controls, (**B**) preHD from controls and (**C**) manHD from preHD groups. sPLS-DA models for assessing the discriminatory potential of all 26 CSF markers for distinguishing (**D–F**) HD mutation carriers from controls, (**G–I**) preHD from controls and (**J–L**) manHD from preHD groups. Two-dimensional score plots showing segregation of (**D**) controls and HD mutation carriers , (**G**) controls and preHD, and (**J**) preHD and manHD. The top 10 CSF proteins ranked based on their relative importance for discriminating (**E**) HD mutation carriers from controls, (**H**) preHD from controls and (**K**) manHD from preHD disease. Grey bars represent the percentage of events a variable was selected by sPLS-DA in the bootstrapped samples. ROC curves generated using sPLS-DA models showing discriminatory ability to distinguish (**F**) HD mutation carriers from controls, (**I**) preHD from controls and (**L**) manHD from preHD individuals. Age-adjusted values were used for all analyses. AUC = area under the curve; manHD = manifest Huntington disease; preHD = premanifest Huntington disease; ROC = receiver operating characteristic; sPLS-DA = sparse partial least squares discriminant analysis.

PENK showed the strongest discriminatory ability of any CSF protein for distinguishing between HD mutations carriers and controls (Fig. 2A, AUC = 0.94, 95% CI: 0.86–1.00, *P* = 0.0003), accurately classifying 79.2% of HD mutation carriers and 100% of control individuals. PENK was also the most discriminant CSF protein for distinguishing preHD from controls (Fig. 2B, AUC = 0.92, 95% CI: 0.78–1.00, *P* = 0.005), correctly classifying 75% of preHD and 100% of control individuals. CHI3L1 showed moderate discriminatory power for distinguishing between manHD and preHD individuals (Fig. 2C, AUC = 0.70, 95% CI: 0.42–0.98, *P* = 0.111), accurately classifying 93.8% of manHD but only 62.5% of preHD individuals.

CSF NEFL was previously shown to have high accuracy for stratifying HD mutation carriers from controls and manHD from preHD individuals.^24^ We observed that NEFL showed a strong discriminatory ability for distinguishing between HD mutation carriers and controls (Supplementary Table 5, AUC = 0.81, 95% CI: 0.62–1.00, *P* = 0.009), but relatively weak discrimination of manHD from preHD subjects in our cohort (Fig. 2C, AUC = 0.69, 95% CI: 0.44–0.94, *P* = 0.142). By comparison, PENK showed a superior discriminatory ability to NEFL for distinguishing between HD mutation carriers and controls, but this did not reach statistical significance (Supplementary Fig. 1, *P* = 0.121).

We next performed sPLS-DA to evaluate the discriminatory potential of combining all 26 CSF proteins. The relative discriminatory importance of individual CSF proteins to each sPLS-DA model is presented in Supplementary Fig. 2.

A two-dimensional score plot segregated HD mutation carriers and controls along components 1 and 2 axes with minimal overlap (Fig. 2D). The model identified PENK (81%), IGHG1 (71.3%), PDYN (60.7%), IGF2 (51.3%) and NEFL (51.1%) as being the five most discriminant CSF proteins for distinguishing between these groups based on the frequency of instances the protein was selected after bootstrapping (Fig. 2E). The ROC curve generated from the sPLS-DA model showed high discriminatory ability for stratifying HD mutation carriers from controls (Fig. 2F, AUC = 0.90, 95% CI: 0.79–1.00, *P* = 0.0006), similar to what was observed with PENK alone (Fig. 2A, AUC = 0.94).

Dimensionality reduction using sPLS-DA segregated individuals from preHD and control groups with minimal overlap (Fig. 2G). The bootstrapped model selected ALB (72.3%), PENK (71.4%), PPP1R1B (70.1%), C1QB (67.3%) and IGHG1 (66.7%) as the five CSF proteins with the highest relative discriminatory importance (Fig. 2H). The ROC curve generated from the sPLS-DA model incorporating all 26 CSF proteins showed high discriminatory performance for stratifying preHD from control individuals (Fig. 2I, AUC = 0.88, 95% CI: 0.69–1.00, *P* = 0.010), similar to PENK alone (Fig. 2B, AUC = 0.92).

The sPLS-DA model for discriminating manHD from preHD also showed strong segregation of subjects on the two-dimensional score plot (Fig. 2J), and identified C4B (82.7%), CTSD (73%), PDYN (67.9%), PENK (67.3%) and CHI3L1 (66.9%) as having the highest relative discriminatory value (Fig. 2K). The ROC curve showed strong discriminatory performance for classifying manHD and preHD groups (Fig. 2L, AUC = 0.95, 95% CI: 0.87–1.00, *P* = 0.003), superior to CHI3L1 alone (Fig. 2C, AUC = 0.70). These findings suggest that the combination of multiple CSF proteins can improve stratification of manHD and preHD individuals.

### Multi-marker CSF protein panels for stratifying subjects based on Huntington disease mutation status and disease severity

We next performed a combinatorial ROC curve analysis using the combiROC analytical tool^58^ to identify marker combinations, comprising the fewest number of CSF proteins (up to five), that could provide the highest discriminatory ability for stratifying individuals based on HD mutation status and disease severity. Each of the most discriminant multi-marker combinations is presented in Supplementary Fig. 3.

**Figure 3:**
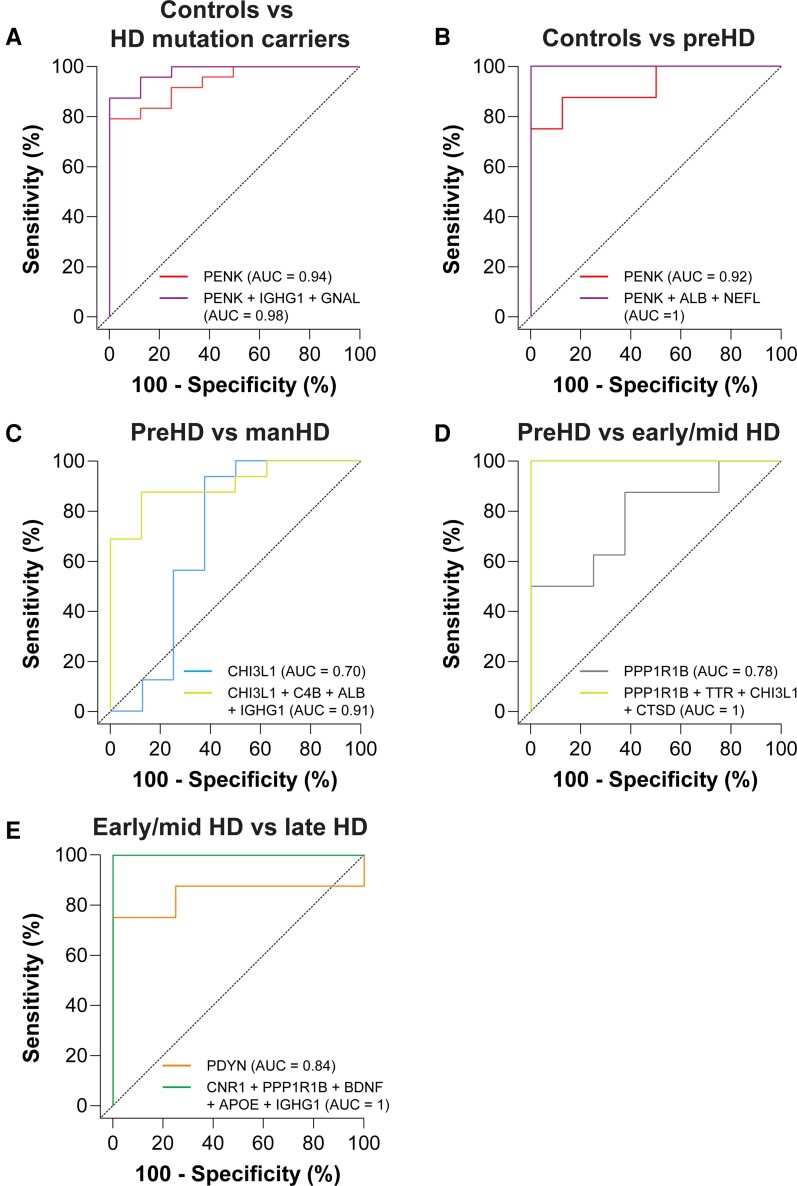
**Multi-marker CSF protein panels for stratifying subjects based on HD mutation status and disease severity.** ROC curves showing individual and combinations of CSF proteins with the greatest discriminatory accuracy for distinguishing between (**A**) HD mutation carriers and controls, (**B**) preHD and controls, (**C**) manHD and preHD, (**D**) early/mid HD and preHD, (**E**) late HD and early/mid HD. Age-adjusted values were used for all analyses. AUC = area under the curve; manHD = manifest Huntington disease; preHD = premanifest Huntington disease; ROC = receiver operating characteristic.

The combination of PENK, IGHG1, and GNAL was able to accurately classify 88% of HD mutation carriers and 100% of control individuals, and improved discriminatory performance (Fig. 3A, AUC = 0.98) beyond what was observed for any individual protein (Fig. 2A, PENK AUC = 0.94) or the combination of all 26 CSF proteins by sPLS-DA (Fig. 2F, AUC = 0.90).

We identified eight unique combinations of three CSF proteins that showed perfect classification of preHD and controls, including combination 3A: PENK, ALB and NEFL (Fig. 3B, AUC = 1). These three marker panels showed superior discriminatory performance compared with PENK alone (Fig. 2B, AUC = 0.92) and the combination of all proteins (Fig. 2I, AUC = 0.88).

The combination of CHI3L1, C4B, IGHG1, and ALB correctly classified 88% of preHD and 88% manHD individuals and showed discriminatory power (Fig. 3C, AUC = 0.91) similar to that observed using all 26 CSF proteins (Fig. 2L, AUC = 0.95).

PPP1R1B showed the highest individual discriminatory accuracy for stratifying early/mid HD and preHD individuals (Supplementary Table 5, AUC = 0.78, 95% CI: 0.55–1.00, *P* = 0.059). Notably, fourteen unique combinations of four CSF proteins, including combination 4A: PPP1R1B, TTR, CHI3L1 and CTSD, showed perfect classification of preHD and early/mid HD individuals (Fig. 3D, AUC = 1).

PDYN showed the highest individual discriminatory ability for distinguishing late HD from early/mid HD individuals (Supplementary Table 4, AUC = 0.84, 95% CI: 0.61–1.00, *P* = 0.021), whereas we identified five unique combinations of five CSF proteins that perfectly classified individuals with late HD and early/mid HD, including combination 5A: CNR1, PPP1R1B, BDNF, APOE and IGHG1 (Fig. 3E, AUC = 1).

## Discussion

In this study, we utilized nanoLC-PRM-MS to quantify levels of 26 proteins in CSF from HD mutation carriers and healthy control individuals. Our primary objective was to replicate previously reported changes in CSF protein markers and to investigate whether novel candidate CSF proteins were altered in HD. Consistent with previous reports, we observed that NEFL,^24,25,27–31^ PENK,^41^ PDYN^42^ and CTSD^38^ were significantly altered in the CSF of HD mutation carriers compared with controls after adjustment for age.

Multiple studies have reproducibly shown increased levels of blood and CSF NEFL in HD.^24,25,27–31^ Elevated levels of NEFL in biofluids have also been reported in other neurological diseases (reviewed in^34^) highlighting its utility as a biomarker of neuronal injury, but one that is not specific to HD. NEFL is currently being used in HD clinical trials as an exploratory biomarker of disease progression and to assess therapeutic efficacy.

We found NEFL levels to be significantly increased in the CSF of late HD subjects compared with control individuals (*P* = 0.002), and trends towards elevated NEFL in early/mid HD compared with controls (*P* = 0.073) and late HD compared with preHD (*P* = 0.068). We did not observe a significant increase of CSF NEFL in manHD compared with the preHD, as reported previously using immunoassays to measure NEFL.^24,27,30^ We did however measure significant associations of CSF NEFL with CAP (*ρ* = 0.44) and SWR scores (*ρ* = −0.50).^24,30^ Our findings support the continued use of NEFL as an exploratory biomarker for monitoring disease severity in clinical trials for HD.

PENK and PDYN are highly expressed in distinct striatal medium spiny neuron (MSN) populations^2^ and are downregulated in the caudate of post-mortem HD brains.^44^ Both PENK and PDYN precursor proteins are cleaved to generate secreted peptides that modulate neurotransmission and regulate various neural functions in the brain. PENK levels in CSF were reported to be decreased in manHD compared with preHD and healthy controls using LC-MS.^41^ We measured a significant reduction of PENK in preHD (*P* = 0.004), early/mid HD (*P* = 0.012) and late HD (*P* = 0.0002) compared with controls and observed moderate correlations with CAP score (*ρ* = −0.48) and SDMT (*ρ* = 0.42).

Reduced CSF PDYN was recently reported in manHD patients compared with controls using targeted LC-MS.^42^ This study found that levels of PDYN were not decreased in other neurodegenerative diseases, including Alzheimer’s disease, Parkinson’s disease, and amyotrophic lateral sclerosis, suggesting that changes of CSF PDYN may be unique to HD.^42^ We found PDYN to be significantly reduced in preHD (*P* = 0.004), early/mid HD (*P* = 0.012) and late HD (*P* = 0.0003) compared with controls. PDYN also showed strong associations with TFC (*ρ* = 0.53), VF (*ρ* = 0.55) and SDMT (*ρ* = 0.57) in manHD individuals. We postulate that reduced CSF PENK and PDYN in preHD individuals may reflect early functional disturbances in the health of MSNs prior to disease onset and differential loss of specific MSN sub-populations at more advanced stages of HD.

CTSD is a lysosomal protease expressed in the brain that has been shown to promote the degradation of mHTT in primary neurons.^60^ Levels of CTSD in the CSF were reported in one study to be significantly decreased in HD mutation carriers by MS^38^ and in another to be unchanged between manHD, preHD and controls using PRM-MS.^61^ Consistent with these reports, we found CTSD to be significantly reduced in the CSF of HD mutation carriers compared with controls (*P* = 0.044) but not significantly changed across disease stages.

In contrast to previous reports, we did not detect significant changes in C1QC,^38^ C4B,^38^ CHI3L1,^30,38,39^ CLU,^40,41^ FAT2^41^ or TTR^38,41,43^ protein levels in the CSF of HD mutation carriers compared with controls. These discordant findings could be due to differences in patient demographics and clinical characteristics, methodology used for the detection of protein analytes in CSF, and/or the specific peptides that were selected for analysis by PRM-MS in our study.

BDNF is a growth factor required for the survival of various neuronal populations in the CNS and is downregulated in the caudate and putamen of post-mortem HD brains compared with age-matched controls.^62^ Levels of BDNF in the CSF were previously reported to be unchanged across HD stages using an immunoassay.^63^ We observed a strong trend towards a reduction of BDNF in late HD compared with controls (*P* = 0.053) and moderate correlations with TFC (*ρ* = 0.45) and SDMT (*ρ* = 0.48). These findings suggest that reduced CSF BDNF may reflect the depletion of BDNF production/release^64^ or even the loss of cortical neurons at advanced stages of HD.^5^ Additional studies to investigate CSF BDNF as a potential biomarker for HD may be warranted.

CSF to blood ALB^65,66^ and IgG quotients,^65^ routinely used to measure blood–brain barrier (BBB)/blood–CSF barrier (BCSFB) dysfunction and intrathecal IgG production, were previously found to be unchanged in the CSF of HD mutation carriers compared with controls. Our data showed a strong trend towards increased ALB in HD mutation carriers compared with controls *(P* = 0.068) and a significant increase of CSF IGHG1 (heavy chain constant domain of IgG) in the late HD compared with controls (*P* = 0.004). The increased CSF albumin and IGHG1 could reflect neurovascular abnormalities and BBB/BCSFB dysfunction which have been reported in HD.^67–69^ Moreover, elevated CSF IGHG1 at advanced stages of HD may suggest increased local CNS IgG synthesis, a marker of CNS inflammation.

In addition to reproducing reported changes in previously investigated CSF biomarkers, we also identified novel candidate CSF proteins whose levels were altered in HD CSF. GNAL, which is highly expressed in the basal ganglia, plays an important role in MSN dopamine signalling.^45,70^ Reduced levels of GNAL have been reported in the caudate and putamen of HD patients.^44,45^ We found GNAL to be significantly elevated in the CSF of HD mutation carriers compared with controls (*P* = 0.043), which could reflect an increased release from degenerating striatal MSNs.

IGF2 is a regulator of neurogenesis, synaptic formation and spine maturation in the brain that plays a role in learning and memory functions.^71–73^ Importantly, reduced IGF2 levels have been reported in the striatum and plasma from HD patients.^50^ We detected significantly elevated IFG2 levels in late HD compared with controls (*P* = 0.012), and moderate correlations with TFC (*ρ* = −0.40), TMS (*ρ* = 0.40), and SWR (*ρ* = −0.42) in manHD individuals. The unexpected increase of CSF IGF2 in HD mutation carriers is consistent with reports from Alzheimer’s disease.^74,75^ We postulate that elevated CSF IGF2 may reflect increased release from IGF2-producing cells (e.g. neural stem cells^73^) or potentially a compensatory neuroprotective mechanism in the brain.

CNR1 is highly expressed in the basal ganglia where it modulates synaptic functions involved in motor behaviour.^76^ Early downregulation of CNR1 has been reported in the striatum of HD patients.^4,44^ Levels of CNR1 were decreased in late HD compared with controls (*P* = 0.055) and preHD (*P* = 0.108) and were significantly reduced in CSF from late HD compared early/mid HD (*P* = 0.008). Moreover, CSF CNR1 levels were strongly correlated with TFC (*ρ* = 0.58), VF (*ρ* = 0.52) and SDMT (*ρ* = 0.56) in manHD. Reduced CSF CNR1 could be a marker that reflects the loss of CNR1-expressing neurons in the basal ganglia at advanced stages of HD.

C1Q (composed of A, B and C polypeptide chains), a component of the complement C1 recognition complex of the classical pathway, is released from CNS cells in response to inflammatory stimuli in neurodegenerative diseases (reviewed in^77^). In HD, upregulation of early complement activators and regulators from reactive microglia has been reported in the striatum of HD patients.^78^ We found CSF C1QB to be modestly increased in early/mid HD compared with preHD and significantly reduced in late HD compared with early/mid HD (*P* = 0.008) and controls (*P* = 0.010). Surprisingly, we did not find C1QC to be significantly altered, although similar trends in levels were observed. C1QB also showed a strong association with SDMT (*ρ* = 0.51) in manHD individuals. These findings suggest that CSF C1QB levels may reflect early HD-associated complement activation in the brain and potential dysregulation of this pathway at more advanced stages of disease.

IDO1, a rate-limiting enzyme in the kynurenine pathway, was reported to be upregulated and have increased activity in the striatum of an HD mouse model.^49^ IDO1 levels were significantly decreased in late HD compared with controls (*P* = 0.020), and showed moderate to strong correlations with CAP score (*ρ* = −0.45), TFC (*ρ* = 0.43) and VF (*ρ* = 0.50). The reduction of IDO1 in the CSF could suggest dysregulation of the kynurenine pathway in the brain or it may be a marker of cell loss in the striatum in lateHD.

Together our data suggest that GNAL, IGF2, CNR1, C1QB and IDO1 may represent promising CSF biomarker candidates that reflect distinct HD-associated pathophysiological alterations in the CNS.

A secondary objective of our study was to compare the discriminatory potential of individual CSF markers and combinations of CSF markers for distinguishing individuals based on HD mutation status and disease severity. We identified PENK and PDYN as being the most discriminant individual CSF proteins for distinguishing HD mutation carriers from controls. Notably, PENK (AUC = 0.94) and PDYN (AUC = 0.84) each showed superior discrimination of HD mutation carriers from controls compared with NEFL alone (AUC = 0.81). Moreover, PENK (AUC = 0.92) also showed the highest discriminatory power for distinguishing preHD from controls. No individual CSF protein showed high discriminatory accuracy for distinguishing between preHD and manHD individuals in our cohort, with CHI3L1 (AUC = 0.70) showing only moderate discriminatory power.

sPLS-DA models incorporating all 26 CSF markers used to stratify either HD mutation carriers and controls (AUC = 0.90) or preHD and controls (AUC = 0.88) showed discriminatory performances similar to PENK alone. In contrast, the combination of all CSF proteins improved the stratification of manHD from preHD (AUC = 0.95) compared with CHI3L1 (AUC = 0.70) or any other individual protein, highlighting the increased discriminatory value of combining multiple CSF markers.

We also performed a combinatorial ROC curve analysis and identified exploratory multi-marker CSF panels with up to five proteins that, in all instances, showed superior discriminatory performance compared with individual proteins for distinguishing individuals based on HD mutation status and disease severity.

The combination of PENK, NEFL and ALB showed perfect discrimination between preHD and control individuals in our cohort suggesting that changes in these CSF proteins represent early events in disease pathogenesis, prior to overt symptomatic onset. Furthermore, all eight lead three marker combinations included PENK, highlighting the importance of this protein for distinguishing between preHD and controls.

The panel consisting of CHI3L1, C4B, IGHG1, and ALB showed high discriminatory power (AUC = 0.91) for distinguishing preHD from manHD individuals, with sensitivity and specificity superior to CHI3L1 alone (AUC = 0.70) and similar to that observed with all 26 CSF markers by sPLS-DA (AUC = 0.95). These data highlight the additive classification performance that is possible even when combining markers that individually have a weak or moderate discriminatory ability.

Moreover, we identified 14 unique 4 marker CSF protein panels that showed perfect discrimination of early/mid HD from preHD individuals, including combinations of C4B, TTR, ALB, and CYCS, as well as PPP1R1B, C4B, TTR and CTSD. Notably, ALB (*r* = 0.75), C4B (*r* = −0.74), CTSD (*r* = 0.66) and TTR (*r* = 0.86) were strongly correlated with years to predicted disease onset in preHD individuals.^10^ We postulate that panels of CSF markers could be used in conjunction with CAG repeat length to improve the accuracy of disease-onset estimates.

Multi-marker CSF protein panels that can accurately discriminate between preHD and early/mid HD or manHD individuals could help define the optimal timing of therapeutic intervention. Preclinical studies in animal models of HD would be needed to assess whether any of the exploratory CSF protein combinations respond to candidate therapies in a manner that suggests therapeutic benefit. If validated, such biomarker panels could provide objective exploratory pharmacodynamic measures for monitoring therapeutic response in preventative trials for HD.

Finally, we identified multiple CSF marker panels, including the combination of CNR1, PPP1R1B, BDNF, APOE and IGHG1, that showed perfect classification of late HD and early/mid HD individuals. This combination of CSF proteins likely reflects alterations in neuronal health, neurotrophic support, lipid metabolism, neuroinflammation, and BBB/BCSFB integrity in the CNS which have been linked with disease severity. Given the complex pathogenesis of HD and associated alterations of numerous biological pathways over the natural history of the disease, it is likely that combinations of molecular biomarkers assessing multiple processes related to HD pathophysiology in parallel will be favoured for use in clinical trials to complement existing clinical and imaging biomarkers. Such panels could provide additional cell-type or pathway-specific resolution into HD-associated pathophysiological changes compared with a single biomarker, such as NEFL, which likely reflects general axonal damage/neuronal injury in the CNS.

MS-based methods are capable of sensitive detection of proteins in biofluids, comparable to other analytical assays, but may provide superior specificity through the identification of multiple specific peptide sequences for any individual protein.^79,80^ Furthermore, targeted MS methods have high multiplexing capability (>100 peptides per assay) which is difficult to achieve with conventional assays (e.g. immunoassays).^81^ Although MS-based assays may not be practical or cost-effective for routine clinical use, the exploratory multi-marker CSF protein panels identified in this study could be used to help guide the design of multiplex immunoassays that would be more amenable to clinical practice. To enable this translation, longitudinal observational studies would be needed to quantify absolute concentrations of individual CSF proteins and define biomarker signatures in HD mutation carriers over the natural history of the disease. We also identified multiple unique CSF protein combinations that are different in composition but that show equivalent discriminatory performance for stratifying subjects based on disease severity which may provide additional flexibility for assay development and could help validation of such assays for clinical use.

The major limitation of our study was the relatively small number of CSF samples analysed. Replication studies with CSF collected from larger cohorts of HD mutation carriers (e.g. HDClarity, NCT02855476) would be needed to validate our exploratory findings. Furthermore, studies to assess whether any of the candidate CSF proteins studied here are also altered in other biofluids (e.g. blood) may be warranted.

In this study, we show compelling evidence to suggest that combinations of CSF markers can outperform individual markers for stratifying individuals based on HD mutation status and disease severity. We postulate that the multi-marker CSF protein panels defined herein may be useful for improving the accuracy of age-of-onset estimates for HD and complement clinical biomarkers for monitoring disease severity.

## Supplementary Material

fcac309_Supplementary_Data

## Data Availability

Data from this study can be made available upon reasonable request.

## Abbreviations

ANCOVA =: analysis of covariance
AUC =: area under the curve
BBB =: blood–brain barrier
BCSFB =: blood–CSF barrier
BMI =: body mass index
CAG =: cytosine–adenine–guanine
CAP =: CAG-age product
CI =: confidence interval
DCL =: diagnostic confidence level
DDA =: data-dependent acquisition
GLM =: general linear model
HCD =: higher-energy collisional dissociation
HD =: Huntington disease
manHD =: manifest Huntington disease
MD =: mean difference
mHTT =: mutant huntingtin
MSN =: medium spiny neuron
nanoLC-PRM-MS =: nanoflow liquid chromatography-coupled parallel-reaction monitoring mass spectrometry
OR =: odds ratio
preHD =: premanifest Huntington disease
ROC =: receiver operating characteristic
SD =: standard deviation
SDMT =: symbol digit modality test
sPLS-DA =: sparse partial least square discriminant analysis
SWR =: Stroop word reading
TFC =: total functional capacity
TMS =: total motor score
UHDRS =: Unified Huntington’s disease rating scale
VF =: verbal fluency
