# Supplementary material for: Cerebrospinal fluid biomarkers for assessing Huntington disease onset and severity
Source: Brain Commun. 2022 Nov 25;4(6):fcac309. doi: 10.1093/braincomms/fcac309 (PMC9746690; doi:10.1093/braincomms/fcac309)
Supplement: fcac309_Supplementary_Data [file fcac309_supplementary_data.zip › Supplementary_figures.pdf]

Supplementary Figure 1: Comparison of NEFL and PENK for discriminating HD mutation carriers from controls. ROC curve and AUC values comparing the discriminatory ability of NEFL and PENK for distinguishing between HD mutation carriers and controls.

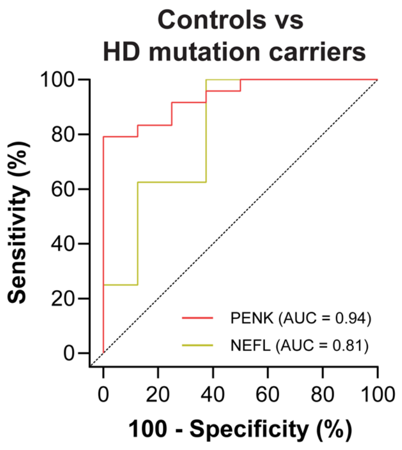

Supplementary Figure 2: Discriminatory importance of CSF proteins for stratification of HD stages. CSF proteins ranked based on their relative importance for discriminating (**A**) HD mutation carriers from controls, (**B**) preHD from controls and (**C**) manHD from preHD groups. Fold changes represent the ratio of age-adjusted means between groups. Grey bars represent the percentage of events a variable was selected by sPLS-DA in the bootstrapped samples.

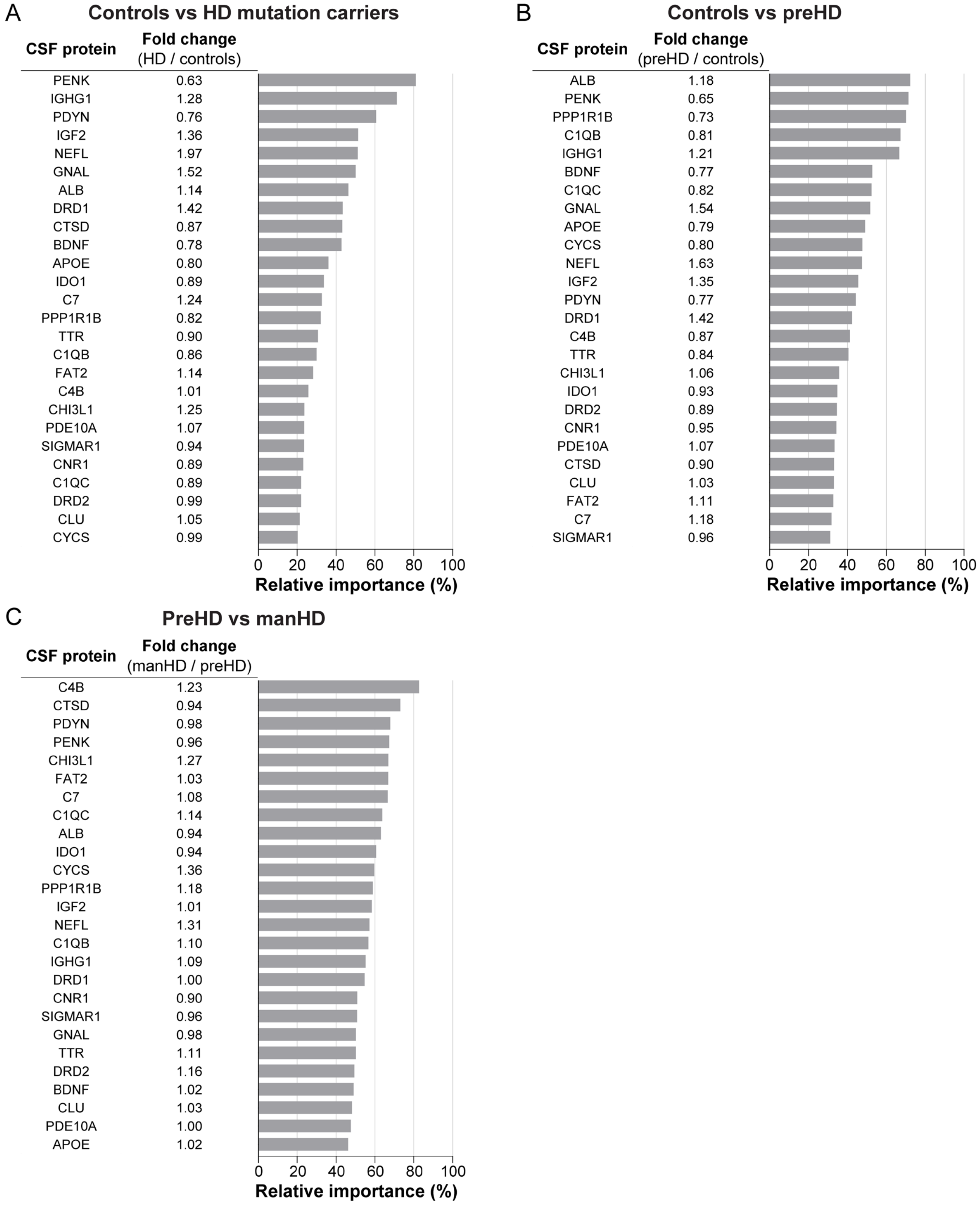

Supplementary Figure 3: Multi-marker CSF protein panels for stratifying subjects based on HD mutation status and disease severity. AUC values of individual and combinations of CSF proteins with the highest discriminatory ability for distinguishing (**A**) HD mutation carriers from controls, (**B**) preHD from controls, (**C**) manHD from preHD, (**D**) early/mid HD from preHD, and (**E**) late HD from early/mid HD.

A

Controls vs HD mutation carriers

1 marker		2 markers				3 markers	
Individual protein		Combination 2A		Combination 2B		Combination 3	
Protein	AUC	Proteins	AUC	Proteins	AUC	Proteins	AUC
PENK	0.94	PENK<br>NEFL	0.96	PENK<br>IGHG1	0.96	PENK<br>GNAL<br>IGHG1	0.98

B

Controls vs preHD

1 marker		2 markers		3 markers															
Individual protein		Combination 2		Combination 3A		Combination 3B		Combination 3C		Combination 3D		Combination 3E		Combination 3F		Combination 3G		Combination 3H	
Protein	AUC	Proteins	AUC	Proteins	AUC	Proteins	AUC	Proteins	AUC	Proteins	AUC	Proteins	AUC	Proteins	AUC	Proteins	AUC	Proteins	AUC
PENK	0.92	PENK<br>ALB	0.98	PENK<br>ALB<br>NEFL	1	PENK<br>ALB<br>IGF2	1	PENK<br>ALB<br>C7	1	PENK<br>ALB<br>BDNF	1	PENK<br>ALB<br>APOE	1	PENK<br>ALB<br>IGHG1	1	PENK<br>IGHG1<br>NEFL	1	PENK<br>IGF2<br>C7	1

C

PreHD vs manHD

1 marker		2 markers		3 markers		4 markers	
Individual protein		Combination 2		Combination 3		Combination 4	
Protein	AUC	Proteins	AUC	Proteins	AUC	Proteins	AUC
CHI3L1	0.70	C4B<br>IGHG1	0.84	C4B<br>IGHG1<br>NEFL	0.87	CHI3L1<br>C4B<br>IGHG1<br>ALB	0.91

D

PreHD vs early/mid HD

1 marker		2 markers		3 markers						4 markers							
Individual protein		Combination 2		Combination 3A		Combination 3B		Combination 3C		Combination 4A		Combination 4B		Combination 4C		Combination 4D	
Protein	AUC	Proteins	AUC	Proteins	AUC	Proteins	AUC	Proteins	AUC	Proteins	AUC	Proteins	AUC	Proteins	AUC	Proteins	AUC
PPP1R1B	0.78	PPP1R1B<br>TTR	0.89	PPP1R1B<br>TTR<br>CHI3L1	0.95	TTR<br>CHI3L1<br>CTSD	0.95	C4B<br>TTR<br>CNR1	0.95	PPP1R1B<br>TTR<br>CHI3L1<br>CTSD	1	PPP1R1B<br>TTR<br>ALB<br>CTSD	1	TTR<br>ALB<br>CTSD<br>CYCS	1	TTR<br>CHI3L1<br>ALB<br>CTSD	1

4 markers																			
Combination 4E		Combination 4F		Combination 4G		Combination 4H		Combination 4I		Combination 4J		Combination 4K		Combination 4L		Combination 4M		Combination 4N	
Proteins	AUC	Proteins	AUC	Proteins	AUC	Proteins	AUC	Proteins	AUC	Proteins	AUC	Proteins	AUC	Proteins	AUC	Proteins	AUC	Proteins	AUC
C4B<br>TTR<br>ALB<br>CYCS	1	PPP1R1B<br>TTR<br>ALB<br>CYCS	1	TTR<br>CHI3L1<br>CTSD<br>CYCS	1	PPP1R1B<br>C4B<br>TTR<br>CTSD	1	TTR<br>CHI3L1<br>CTSD<br>CNR1	1	C4B<br>TTR<br>CTSD<br>CNR1	1	PPP1R1B<br>C1QB<br>TTR<br>CTSD	1	C1QB<br>TTR<br>CTSD<br>CYCS	1	C1QB<br>TTR<br>CTSD<br>CNR1	1	C1QB<br>TTR<br>CHI3L1<br>CTSD	1

E

Early/mid HD vs late HD

1 marker		2 markers		3 markers								4 markers		5 markers			
Individual protein		Combination 2		Combination 3A		Combination 3B		Combination 3C		Combination 3D		Combination 4		Combination 5A		Combination 5B	
Protein	AUC	Proteins	AUC	Proteins	AUC	Proteins	AUC	Proteins	AUC	Proteins	AUC	Proteins	AUC	Proteins	AUC	Proteins	AUC
PDYN	0.84	PDYN<br>PENK	0.88	PDYN<br>PENK<br>IGHG1	0.91	PDYN<br>C1QB<br>IGHG1	0.91	PENK<br>CNR1<br>IGF2	0.91	CNR1<br>C1QB<br>IGHG1	0.91	CNR1<br>PPP1R1B<br>APOE<br>IGHG1	0.97	CNR1<br>PPP1R1B<br>BDNF<br>APOE<br>IGHG1	1	CNR1<br>BDNF<br>APOE<br>IGF2<br>IDO1	1

5 markers					
Combination 5C		Combination 5D		Combination 5E	
Proteins	AUC	Proteins	AUC	Proteins	AUC
CNR1<br>BDNF<br>C1QB<br>IGF2<br>IDO1	1	CNR1<br>PPP1R1B<br>BDNF<br>C1QB<br>IGHG1	1	PDYN<br>CNR1<br>PPP1R1B<br>C1QB<br>IGHG1	1
