# Supplementary material for: Cerebrospinal fluid biomarkers for assessing Huntington disease onset and severity
Source: Brain Commun. 2022 Nov 25;4(6):fcac309. doi: 10.1093/braincomms/fcac309 (PMC9746690; doi:10.1093/braincomms/fcac309)
Supplement: fcac309_Supplementary_Data [file fcac309_supplementary_data.zip › Supplementary_tables.pdf]

Supplementary Table 1: Peptide sequences and correlations between individual peptides measured by nanoLC-PRM-MS. Summary of peptide sequences measured by nanoLC-PRM-MS and correlations between unadjusted peptide values for each of the 26 CSF protein candidates in all participants. Pearson's correlation coefficients (r) are presented.

Protein	Peptide; Charge	Peptide #	1	2	3	4	5	6	7	8	9	10	11	12	13	14	15
ALB	DLGEENFK;2	1	1.00	0.68	0.83	0.69	0.73	0.74	0.80	0.74	0.86	0.75	0.59	0.74			
	ETYGEMADC[+57]C[+57]AK;2	2	0.68	1.00	0.79	0.51	0.70	0.66	0.60	0.73	0.76	0.69	0.68	0.68			
	AAFTEC[+57]C[+57]QAADK;2	3	0.83	0.79	1.00	0.65	0.93	0.81	0.80	0.82	0.87	0.87	0.73	0.84			
	VHTEC[+57]C[+57]HGDLLEC[+57]ADDR;3	4	0.69	0.51	0.65	1.00	0.66	0.53	0.78	0.52	0.80	0.53	0.66	0.68			
	YIC[+57]ENQDSISSK;2	5	0.73	0.70	0.93	0.66	1.00	0.81	0.76	0.73	0.81	0.87	0.71	0.80			
	TYETTLEK;2	6	0.74	0.66	0.81	0.53	0.81	1.00	0.85	0.65	0.71	0.90	0.58	0.71			
	C[+57]C[+57]AAADPHEC[+57]YAK;3	7	0.80	0.60	0.80	0.78	0.76	0.85	1.00	0.64	0.81	0.77	0.65	0.73			
	QNC[+57]ELFEQLGEYK;2	8	0.74	0.73	0.82	0.52	0.73	0.65	0.64	1.00	0.87	0.72	0.75	0.90			
	VPQVSTPTLVEVSR;2	9	0.86	0.76	0.87	0.80	0.81	0.71	0.81	0.87	1.00	0.79	0.81	0.91			
	C[+57]C[+57]TESLVNR;2	10	0.75	0.69	0.87	0.53	0.87	0.90	0.77	0.72	0.79	1.00	0.66	0.78			
	PC[+57]FSALEVDETYVPK;2	11	0.59	0.68	0.73	0.66	0.71	0.58	0.65	0.75	0.81	0.66	1.00	0.85			
	AVMDDFAAFVEK;2	12	0.74	0.68	0.84	0.68	0.80	0.71	0.73	0.90	0.91	0.78	0.85	1.00			
APOE	SELEEQLTPVAEETR;2	1	1.00	0.88	0.91	0.94											
	AATVGSLAGQPLQER;2	2	0.88	1.00	0.80	0.88											
	SWFEPLVEDMQR;2	3	0.91	0.80	1.00	0.87											
	VQAAVGTSAAPVPSDNH;2	4	0.94	0.88	0.87	1.00											
BDNF	IDTSCVCTLTIK;2	1	1.00	0.77													
	THGTLESVNGPK;2	2	0.77	1.00													
C1QB	LEQGENVFLQATDK;2	1	1.00	0.73													
	VITNANENYEPR;2	2	0.73	1.00													
C1QC	FQSVFTVTR;2	1	1.00	0.68	0.76	0.64											
	QTHQPPAPNSLIR;3	2	0.68	1.00	0.93	0.75											
	TNQVNSGGVLLR;2	3	0.76	0.93	1.00	0.78											
	FNAVLTNPQGDYDTSTGK;2	4	0.64	0.75	0.78	1.00											
C4B	VDFTLSSER;2	1	1.00	0.85	0.45	0.78	0.87	0.72	0.91	0.87	0.94	0.80	0.91	0.94	0.94	0.93	0.85
	SC[+57]GLHQLLR;2	2	0.85	1.00	0.73	0.76	0.85	0.82	0.74	0.87	0.77	0.85	0.87	0.82	0.89	0.83	0.89
	GPEVQLVAHSPWLK;3	3	0.45	0.73	1.00	0.46	0.54	0.70	0.38	0.51	0.32	0.67	0.52	0.46	0.51	0.36	0.62
	YVLPNFEVK;2	4	0.78	0.76	0.46	1.00	0.88	0.80	0.74	0.86	0.79	0.63	0.89	0.69	0.80	0.65	0.91
	PVQGVAYVR;2	5	0.87	0.85	0.54	0.88	1.00	0.82	0.80	0.85	0.85	0.72	0.93	0.78	0.86	0.77	0.90
	LVNGQSHISLSK;3	6	0.72	0.82	0.70	0.80	0.82	1.00	0.62	0.84	0.67	0.76	0.83	0.67	0.80	0.65	0.88
	AEFQDALEK;2	7	0.91	0.74	0.38	0.74	0.80	0.62	1.00	0.80	0.97	0.63	0.85	0.93	0.90	0.88	0.75
	LNMGITDLQGLR;2	8	0.87	0.87	0.51	0.86	0.85	0.84	0.80	1.00	0.86	0.82	0.90	0.81	0.93	0.82	0.87
	EMSGSPASGIPVK;2	9	0.94	0.77	0.32	0.79	0.85	0.67	0.97	0.86	1.00	0.70	0.88	0.92	0.93	0.89	0.78
	VGDTLNLNLR;2	10	0.80	0.85	0.67	0.63	0.72	0.76	0.63	0.82	0.70	1.00	0.75	0.80	0.78	0.77	0.76
	GQIVFMNR;2	11	0.91	0.87	0.52	0.89	0.93	0.83	0.85	0.90	0.88	0.75	1.00	0.83	0.91	0.82	0.91
	LELSVDGAK;2	12	0.94	0.82	0.46	0.69	0.78	0.67	0.93	0.81	0.92	0.80	0.83	1.00	0.92	0.91	0.78
	GLC[+57]VATPVQLR;2	13	0.94	0.89	0.51	0.80	0.86	0.80	0.90	0.93	0.93	0.78	0.91	0.92	1.00	0.88	0.86
	VLSLAQEQVGGSPEK;2	14	0.93	0.83	0.36	0.65	0.77	0.65	0.88	0.82	0.89	0.77	0.82	0.91	0.88	1.00	0.77
	QGSFQGGFR;2	15	0.85	0.89	0.62	0.91	0.90	0.88	0.75	0.87	0.78	0.76	0.91	0.78	0.86	0.77	1.00
C7	VLFYVDSEK;2	1	1.00	0.75	0.68	0.63	0.44	0.97	0.90	0.97							
	LTPLYELVK;2	2	0.75	1.00	0.63	0.37	0.40	0.63	0.70	0.65							
	DGFVQDEGTMFPVGK;2	3	0.68	0.63	1.00	0.31	0.59	0.62	0.60	0.64							
	IAC[+57]VLPVLMDGIQSHPQK;2	4	0.63	0.37	0.31	1.00	0.31	0.63	0.73	0.64							
	ENPLTQAVPK;2	5	0.44	0.40	0.59	0.31	1.00	0.32	0.28	0.39							
	MPYEC[+57]GPSLDVC[+57]AQDER;2	6	0.97	0.63	0.62	0.63	0.32	1.00	0.91	0.98							
	ILPLTVC[+57]K;2	7	0.90	0.70	0.60	0.73	0.28	0.91	1.00	0.91							
	EQTMSEC[+57]EAGALR;2	8	0.97	0.65	0.64	0.64	0.39	0.98	0.91	1.00							
CHI3L1	EGDGSC[+57]FPDALDR;2	1	1.00	0.73	0.85												
	EAGTLAYYEIC[+57]DFLR;2	2	0.73	1.00	0.84												
	FPLTNAIK;2	3	0.85	0.84	1.00												
CLU	EIQNAVNGVK;2	1	1.00	0.92	0.90	0.84	0.86	0.90									
	TLLSNLEEAK;2	2	0.92	1.00	0.93	0.92	0.94	0.87									
	IDSLLENDR;2	3	0.90	0.93	1.00	0.90	0.91	0.84									
	EILSVDC[+57]STNNPSQAK;2	4	0.84	0.92	0.90	1.00	0.91	0.83									
	ELDESLQVAER;2	5	0.86	0.94	0.91	0.91	1.00	0.89									
	FMETVAEK;2	6	0.90	0.87	0.84	0.83	0.89	1.00									
CNR1	SIIIHTSEDGK;2	1	1.00	0.47													
	YISIHRPLAYK;2	2	0.47	1.00													
CTSD	TMSEVGGSVEDLIAK;2	1	1.00	0.75	0.94												
	FDGILGMAYPR;2	2	0.75	1.00	0.84												
	VSTLPAITLK;2	3	0.94	0.84	1.00												
CYCS	ADLIAYLK;2	1	1.00	0.96													
	TGPNLHGLFGR;2	2	0.96	1.00													
DRD1	YWAISSPFRYER;2	1	1.00	0.70													
	EEAAGIARPLEK;2	2	0.70	1.00													
DRD2	IFEIQTMPNGKTR;2	1	1.00	0.91													
	AFLKILHC;2	2	0.91	1.00													
FAT2	SIMTMSAIDVDELQNLK;2	1	1.00	0.66	0.73	0.67	0.79	0.69	0.65								
	VPQDTVPGVELLR;2	2	0.66	1.00	0.71	0.62	0.80	0.47	0.55								
	STFVGQISEAAPLYSMIMDK;3	3	0.73	0.71	1.00	0.65	0.78	0.71	0.69								
	ILEPEALK;2	4	0.67	0.62	0.65	1.00	0.72	0.77	0.62								
	ASDGLYQDTALVK;3	5	0.79	0.80	0.78	0.72	1.00	0.67	0.69								
	TGVLTVTGPLDYESK;2	6	0.69	0.47	0.71	0.77	0.67	1.00	0.61								
	DPQDTLTYSLAEEETLGR;3	7	0.65	0.55	0.69	0.62	0.69	0.61	1.00								
GNAL	LWDDEGVK;2	1	1.00	0.72	0.51												
	IDSVSLVDYTPTDQDLLR;2	2	0.72	1.00	0.51												
	VNFHMFDVGGQR;2	3	0.51	0.51	1.00												
IDO1	NFLCSLESNPSVR;2	1	1.00	0.69	0.70												
	HLPDLIESGQLR;2	2	0.69	1.00	0.85												
	YILIPASQQPK;2	3	0.70	0.85	1.00												
IGF2	GIVEECCFR;2	1	1.00	0.73	0.57	0.63											
	SCDLALLETYCATPAK;2	2	0.73	1.00	0.57	0.74											
	GIVEEC[+57]C[+57]FR;2	3	0.57	0.57	1.00	0.88											
	SC[+57]DLALLETYC[+57]ATPAK;2	4	0.63	0.74	0.88	1.00											
IGHG1	TPEVTC[+57]VVVDVSHEDPEVK;3	1	1.00	0.81	0.83	0.64	0.91	0.39									
	FNWYVDGVEVHNAK;3	2	0.81	1.00	0.74	0.43	0.77	0.34									
	ALPAPIEK;2	3	0.83	0.74	1.00	0.75	0.82	0.51									
	NQVSLTC[+57]LVK;2	4	0.64	0.43	0.75	1.00	0.62	0.24									
	TTPPVLDSDGSFFLYSK;2	5	0.91	0.77	0.82	0.62	1.00	0.54									
	SLSLSPGK;2	6	0.39	0.34	0.51	0.24	0.54	1.00									
NEFL	EYQDLLNVK;2	1	1.00	0.84	0.82	0.94											
	LSFTSVGSITSGYSQSSQVFGR;2	2	0.84	1.00	0.80	0.83											
	VEGAGEEQAAK;2	3	0.82	0.80	1.00	0.83											
	FTVLTESAAK;2	4	0.94	0.83	0.83	1.00											
PDE10A	QTELNDFLLDVSK;2	1	1.00	0.58	0.76	0.47											
	TGEVLNIPDAYADPR;2	2	0.58	1.00	0.55	0.48											
	EVDLYTGYTTR;2	3	0.76	0.55	1.00	0.63											
	AIIATDLALYFGNR;2	4	0.47	0.48	0.63	1.00											
PDYN	FLPSISTK;2	1	1.00	0.57	0.69												
	SVGEGPYSELAK;2	2	0.57	1.00	0.92												
	ENTLSKSLEEK;2	3	0.69	0.92	1.00												
PENK	DAEEDDSLANSSDLLKELLETGDNR;3	1	1.00	0.71	0.80												
	DAEEDDSLANSSDLLK;2	2	0.71	1.00	0.96												
	ELLETGDNR;3	3	0.80	0.96	1.00												
PPP1R1B	ASGEGHHLK;2	1	1.00	0.68													
	RPNPCAYTPPSLK;2	2	0.68	1.00													
SIGMAR1	YWAEISDTIISGTFHQWR;2	1	1.00	0.61	0.50												
	EEIAQLAR;2	2	0.61	1.00	0.84												
	LIVELR;2	3	0.50	0.84	1.00												
TTR	CPLMVK;2	1	1.00	0.77	0.75												
	GSPAINVAVHVFR;2	2	0.77	1.00	0.65												
	AADDTWEPFASGK;2	3	0.75	0.65	1.00												

r: 1 – 0.5 – 0

Supplementary Table 2: Comparison of CSF protein levels across disease stages. Fold changes and comparisons of age-adjusted CSF protein levels between controls, preHD, early/mid HD, and late HD individuals. Intergroup differences were assessed using ANCOVA including age as a covariate followed by posthoc analysis using Tukey's test to correct for multiple comparisons. Fold changes represent the ratio of age-adjusted means between groups. *P*-values <0.05 are shown in bold. *Candidates not previously investigated in HD CSF.

			Controls vs preHD		Controls vs early/mid HD		Controls vs late HD		PreHD vs early/mid HD		PreHD vs late HD		Early/mid HD vs late HD	
CSF protein	ANCOVA $F_{(3, 28)}$	ANCOVA *P*-value	Fold change (preHD / controls)	*P*-value	Fold change (early/mid HD / controls)	*P*-value	Fold change (late HD / controls)	*P*-value	Fold change (early/mid HD / preHD)	*P*-value	Fold change (late HD / preHD)	*P*-value	Fold change (late HD / early/mid HD)	*P*-value
ALB	1.611	0.209	1.18	0.156	1.11	0.589	1.12	0.532	0.94	0.804	0.94	0.850	1.01	1.000
APOE*	1.296	0.295	0.79	0.546	0.90	0.925	0.71	0.280	1.14	0.888	0.90	0.960	0.79	0.623
BDNF	2.879	0.054	0.77	0.263	0.91	0.883	0.67	0.053	1.18	0.669	0.87	0.836	0.73	0.221
C1QB*	5.618	**0.004**	0.81	0.240	1.01	1.000	0.77	**0.010**	1.25	0.204	0.95	0.418	0.76	**0.008**
C1QC	0.290	0.832	0.82	0.882	1.00	1.000	0.86	0.944	1.23	0.880	1.06	0.998	0.86	0.943
C4B	0.709	0.555	0.87	0.925	1.17	0.842	0.99	1.000	1.33	0.484	1.13	0.945	0.85	0.809
C7*	1.473	0.243	1.18	0.741	1.18	0.733	1.37	0.177	1.00	1.000	1.16	0.701	1.16	0.710
CHI3L1	0.599	0.621	1.06	0.998	1.41	0.642	1.28	0.856	1.33	0.750	1.20	0.927	0.90	0.980
CLU	0.057	0.982	1.03	0.998	1.08	0.976	1.04	0.996	1.04	0.996	1.01	1.000	0.97	0.998
CNR1*	4.529	**0.010**	0.95	0.988	1.13	0.847	0.58	0.055	1.19	0.666	0.61	0.108	0.52	**0.008**
CTSD	2.167	0.114	0.90	0.503	0.86	0.222	0.83	0.104	0.96	0.943	0.93	0.766	0.97	0.976
CYCS*	0.518	0.673	0.80	0.933	1.22	0.920	0.95	0.999	1.53	0.614	1.19	0.971	0.78	0.859
DRD1*	1.338	0.282	1.42	0.372	1.40	0.421	1.44	0.344	0.98	1.000	1.01	1.000	1.03	0.999
DRD2*	0.337	0.799	0.89	0.955	1.10	0.963	0.97	0.999	1.23	0.753	1.09	0.979	0.89	0.930
FAT2	0.592	0.625	1.11	0.841	1.12	0.814	1.18	0.570	1.01	1.000	1.06	0.964	1.05	0.975
GNAL*	1.942	0.146	1.54	0.238	1.40	0.495	1.62	0.143	0.91	0.957	1.05	0.992	1.16	0.857
IDO1*	3.403	**0.032**	0.93	0.702	0.96	0.431	0.78	**0.020**	1.04	0.961	0.85	0.194	0.81	0.454
IGF2*	4.036	**0.017**	1.35	0.114	1.21	0.496	1.50	**0.012**	0.90	0.799	1.11	0.745	1.24	0.242
IGHG1	4.847	**0.008**	1.21	0.222	1.23	0.151	1.40	**0.004**	1.02	0.996	1.16	0.283	1.14	0.389
NEFL	3.770	**0.022**	1.63	0.162	1.82	0.073	2.47	**0.002**	1.11	0.675	1.51	0.068	1.36	0.151
PDE10A*	0.078	0.972	1.07	0.976	1.06	0.984	1.07	0.977	0.99	1.000	1.00	1.000	1.01	1.000
PDYN	8.391	**0.0004**	0.77	**0.004**	0.81	**0.012**	0.70	**0.0003**	1.05	0.959	0.90	0.780	0.86	0.484
PENK	8.918	**0.0003**	0.65	**0.004**	0.70	**0.012**	0.55	**0.0002**	1.07	0.962	0.85	0.714	0.80	0.424
PPP1R1B*	3.113	**0.042**	0.73	0.338	1.06	0.978	0.67	0.167	1.45	0.176	0.91	0.973	0.63	0.076
SIGMAR1*	0.288	0.834	0.96	0.995	0.99	1.000	0.86	0.836	1.03	0.998	0.90	0.933	0.87	0.871
TTR	1.506	0.235	0.84	0.555	1.03	0.996	0.82	0.490	1.23	0.422	0.98	1.000	0.80	0.363

Supplementary Table 3: Correlation of CSF proteins with years to predicted disease-onset. The relationship of unadjusted CSF protein levels with years to predicted disease-onset were evaluated in preHD mutation carriers using Pearson's correlation. Correlation coefficients (r) are presented and colour scales highlight positive (blue) and negative (red) correlations. Correlations with *P*-values <0.05 are shown in bold.

	Years to predicted disease-onset	
	r	*P*-value
ALB	**0.75**	**0.03**
APOE	-0.06	0.88
BDNF	-0.19	0.65
C1QB	-0.23	0.58
C1QC	-0.39	0.33
C4B	**-0.74**	**0.04**
C7	-0.42	0.30
CHI3L1	-0.42	0.30
CLU	-0.15	0.73
CNR1	-0.11	0.79
CTSD	0.66	0.08
CYCS	-0.20	0.63
DRD1	0.38	0.35
DRD2	0.55	0.16
FAT2	-0.51	0.20
GNAL	0.30	0.48
IDO1	0.19	0.65
IGF2	-0.37	0.36
IGHG1	**0.85**	**0.01**
NEFL	0.09	0.83
PDE10A	0.06	0.88
PDYN	-0.11	0.80
PENK	0.36	0.38
PPP1R1B	-0.02	0.96
SIGMAR1	0.34	0.41
TTR	**0.86**	**0.01**

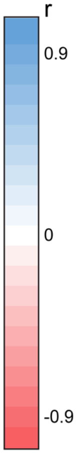

Supplementary Table 4: Correlations between CSF proteins in HD mutation carriers. Associations between age-adjusted CSF protein levels were performed in all HD mutation carriers using Pearson's partial correlation including age as a covariate. Proteins were grouped according to their association with biological processes related to neuronal function, motor behaviour, cognition and memory, synapse organization and plasticity, complement pathway activation, immune response, and apoptosis and cell death. Functional enrichment analysis of CSF proteins was performed using g:GOSt (g:Profiler). Correlation coefficients (r) are presented and colour scales highlight positive (blue) and negative (red) correlations. Correlations with *P*-values <0.05 are shown in bold.

	DRD2	DRD1	CNR1	PPP1R1B	PDE10A	PENK	APOE	BDNF	PDYN	NEFL	SIGMAR1	GNAL	IGF2	C1QB	C1QC	CLU	C4B	C7	CHI3L1	CTSD	IDO1	IGHG1	ALB	CYCS	FAT2
Apoptosis and cell death	■		■				■	■		■						■			■	■	■		■	■	
Immune response	■	■	■				■							■	■	■	■	■	■	■	■	■			
Complement activation														■	■	■	■	■							
Synapse org. and plasticity	■	■			■		■	■		■	■		■	■	■										
Cognition and memory	■	■	■	■			■	■	■				■												
Motor behaviour	■	■	■	■	■	■																			
Neuronal function	■	■	■	■	■	■	■	■	■	■	■	■													

r: 0.9 — 0 — -0.4

	DRD2	DRD1	CNR1	PPP1R1B	PDE10A	PENK	APOE	BDNF	PDYN	NEFL	SIGMAR1	GNAL	IGF2	C1QB	C1QC	CLU	C4B	C7	CHI3L1	CTSD	IDO1	IGHG1	ALB	CYCS	FAT2
DRD1	0.24																								
CNR1	**0.43**	0.19																							
PPP1R1B	**0.62**	0.21	**0.76**																						
PDE10A	**0.54**	**0.46**	0.34	**0.58**																					
PENK	0.02	-0.04	**0.68**	0.36	0.01																				
APOE	0.02	-0.17	**0.61**	**0.43**	-0.11	**0.65**																			
BDNF	0.14	0.00	**0.74**	**0.59**	-0.06	**0.70**	**0.85**																		
PDYN	0.06	0.06	**0.60**	0.38	0.02	**0.54**	**0.64**	**0.65**																	
NEFL	0.08	0.33	-0.05	-0.15	-0.13	-0.14	-0.21	-0.06	-0.04																
SIGMAR1	0.31	**0.51**	0.22	**0.43**	**0.67**	-0.17	-0.36	-0.25	-0.13	-0.04															
GNAL	0.02	**0.53**	-0.04	0.14	**0.51**	-0.11	-0.33	-0.19	**-0.42**	0.12	**0.57**														
IGF2	-0.14	0.07	-0.21	-0.27	-0.05	-0.36	-0.31	-0.37	-0.40	0.10	0.15	0.14													
C1QB	0.18	-0.20	**0.65**	**0.47**	-0.14	**0.60**	**0.81**	**0.82**	**0.48**	-0.17	**-0.41**	-0.34	-0.10												
C1QC	-0.07	-0.30	**0.49**	0.26	-0.22	**0.60**	**0.83**	**0.71**	0.39	-0.23	-0.36	-0.27	0.07	**0.85**											
CLU	0.08	-0.28	**0.47**	0.34	-0.21	**0.48**	**0.92**	**0.78**	**0.53**	-0.17	**-0.46**	**-0.45**	-0.22	**0.80**	**0.80**										
C4B	0.10	-0.22	-0.16	0.21	0.02	-0.40	0.04	0.13	0.07	-0.13	-0.08	-0.18	-0.15	0.11	0.00	0.05									
C7	0.16	-0.01	0.15	0.04	-0.05	-0.07	0.12	0.05	-0.06	0.15	-0.02	-0.10	**0.77**	0.30	0.38	0.20	-0.06								
CHI3L1	0.26	-0.32	0.36	0.39	0.05	0.10	**0.68**	**0.53**	**0.48**	-0.28	-0.27	**-0.44**	-0.17	**0.59**	**0.55**	**0.71**	**0.47**	0.25							
CTSD	0.08	-0.16	0.37	0.32	-0.20	**0.43**	**0.63**	**0.56**	0.35	-0.19	-0.13	-0.16	-0.22	**0.54**	**0.57**	**0.72**	-0.10	-0.03	0.31						
IDO1	0.34	0.12	**0.75**	**0.65**	**0.43**	0.36	0.28	0.35	**0.47**	-0.09	**0.47**	0.02	-0.08	0.29	0.23	0.18	-0.14	0.04	0.26	0.26					
IGHG1	0.01	0.23	-0.26	**-0.45**	-0.10	-0.06	-0.32	-0.39	-0.20	**0.44**	0.09	0.08	0.28	-0.35	-0.15	-0.30	**-0.45**	0.29	-0.38	-0.16	-0.21				
ALB	-0.17	0.38	-0.01	-0.11	0.31	0.11	-0.18	-0.18	-0.15	0.13	**0.50**	**0.61**	-0.05	**-0.45**	-0.21	-0.37	-0.35	-0.12	**-0.44**	-0.07	0.04	**0.50**			
CYCS	0.28	-0.03	**0.51**	**0.53**	0.14	**0.43**	**0.70**	**0.67**	0.34	-0.09	-0.09	-0.07	-0.11	**0.58**	**0.58**	**0.66**	0.16	0.28	**0.53**	**0.48**	0.12	-0.18	-0.04		
FAT2	-0.10	0.14	0.17	0.15	0.12	0.03	0.19	0.14	-0.01	-0.13	0.21	0.08	**0.73**	0.27	0.40	0.16	0.08	**0.74**	0.17	0.01	0.10	0.02	0.03	0.34	
TTR	0.05	0.18	-0.15	-0.07	-0.10	-0.19	-0.22	-0.17	-0.33	0.16	0.15	0.36	-0.05	-0.05	-0.09	-0.18	-0.02	-0.19	-0.33	0.34	-0.18	0.21	0.23	-0.13	-0.25

Supplementary Table 5: Discriminatory power of individual CSF markers for stratification of disease stages. ROC curve analysis evaluating the discriminatory performance of individual CSF protein for distinguishing subjects based on HD mutation status and disease severity. Age-adjusted values were used to generate ROC curves. *P*-values <0.05 are shown in bold.

	Controls vs HD mutation carriers			Controls vs preHD			PreHD vs manHD			PreHD vs early/mid HD			Early/mid HD vs late HD		
**CSF protein**	**AUC**	**95% CI**	***P*-value**	**AUC**	**95% CI**	***P*-value**	**AUC**	**95% CI**	***P*-value**	**AUC**	**95% CI**	***P*-value**	**AUC**	**95% CI**	***P*-value**
ALB	0.74	0.51 to 0.97	**0.045**	0.77	0.51 to 1.00	0.074	0.63	0.40 to 0.85	0.327	0.66	0.37 to 0.94	0.294	0.50	0.20 to 0.80	>0.999
APOE	0.73	0.54 to 0.92	0.056	0.75	0.50 to 1.00	0.093	0.52	0.27 to 0.77	0.854	0.61	0.32 to 0.89	0.462	0.75	0.49 to 1.00	0.093
BDNF	0.73	0.54 to 0.93	0.050	0.75	0.49 to 1.00	0.093	0.54	0.29 to 0.79	0.760	0.63	0.34 to 0.91	0.401	0.78	0.52 to 1.00	0.059
C1QB	0.69	0.51 to 0.87	0.107	0.75	0.50 to 1.00	0.093	0.55	0.32 to 0.79	0.668	0.73	0.48 to 0.99	0.115	0.77	0.50 to 1.00	0.074
C1QC	0.60	0.40 to 0.80	0.408	0.63	0.33 to 0.92	0.401	0.52	0.27 to 0.78	0.854	0.59	0.31 to 0.88	0.529	0.59	0.30 to 0.89	0.529
C4B	0.51	0.26 to 0.76	0.931	0.59	0.30 to 0.88	0.529	0.65	0.40 to 0.90	0.245	0.73	0.47 to 1.00	0.115	0.64	0.34 to 0.94	0.345
C7	0.73	0.52 to 0.93	0.056	0.77	0.51 to 1.00	0.074	0.55	0.32 to 0.78	0.713	0.53	0.22 to 0.84	0.834	0.63	0.34 to 0.91	0.401
CHI3L1	0.55	0.35 to 0.74	0.695	0.64	0.32 to 0.97	0.345	0.70	0.42 to 0.98	0.111	0.72	0.43 to 1.00	0.142	0.64	0.36 to 0.93	0.345
CLU	0.53	0.32 to 0.74	0.794	0.59	0.29 to 0.90	0.529	0.56	0.29 to 0.83	0.624	0.55	0.24 to 0.85	0.753	0.69	0.41 to 0.96	0.208
CNR1	0.60	0.41 to 0.80	0.384	0.61	0.32 to 0.90	0.462	0.58	0.35 to 0.81	0.540	0.66	0.37 to 0.94	0.294	0.83	0.61 to 1.00	**0.027**
CTSD	0.78	0.61 to 0.93	**0.021**	0.64	0.36 to 0.92	0.345	0.69	0.44 to 0.94	0.142	0.66	0.37 to 0.94	0.294	0.66	0.37 to 0.94	0.294
CYCS	0.57	0.34 to 0.79	0.572	0.61	0.32 to 0.90	0.462	0.55	0.32 to 0.79	0.668	0.64	0.36 to 0.92	0.345	0.64	0.36 to 0.93	0.345
DRD1	0.72	0.52 to 0.92	0.068	0.69	0.42 to 0.96	0.208	0.51	0.24 to 0.77	0.951	0.52	0.22 to 0.81	0.916	0.56	0.26 to 0.86	0.674
DRD2	0.59	0.36 to 0.82	0.459	0.64	0.35 to 0.93	0.345	0.59	0.35 to 0.84	0.462	0.58	0.28 to 0.87	0.600	0.53	0.21 to 0.85	0.834
FAT2	0.67	0.43 to 0.91	0.151	0.73	0.45 to 1.00	0.115	0.59	0.37 to 0.82	0.462	0.59	0.29 to 0.90	0.529	0.52	0.22 to 0.81	0.916
GNAL	0.77	0.58 to 0.95	**0.027**	0.72	0.46 to 0.97	0.142	0.53	0.23 to 0.83	0.807	0.52	0.20 to 0.83	0.916	0.61	0.32 to 0.89	0.462
IDO1	0.71	0.48 to 0.94	0.082	0.67	0.39 to 0.95	0.248	0.62	0.38 to 0.85	0.220	0.50	0.20 to 0.80	>0.999	0.72	0.46 to 0.98	0.142
IGF2	0.78	0.61 to 0.95	**0.019**	0.84	0.65 to 1.00	**0.021**	0.51	0.26 to 0.75	0.951	0.64	0.36 to 0.92	0.345	0.75	0.51 to 0.99	0.093
IGHG1	0.83	0.67 to 0.98	**0.006**	0.77	0.53 to 1.00	0.074	0.65	0.42 to 0.87	0.245	0.53	0.23 to 0.83	0.834	0.73	0.48 to 0.99	0.115
NEFL	0.81	0.62 to 1.00	**0.009**	0.77	0.51 to 1.00	0.074	0.69	0.44 to 0.94	0.142	0.63	0.33 to 0.92	0.401	0.69	0.41 to 0.96	0.208
PDE10A	0.53	0.28 to 0.78	0.794	0.55	0.24 to 0.85	0.753	0.58	0.33 to 0.82	0.540	0.58	0.28 to 0.88	0.600	0.52	0.22 to 0.81	0.916
PDYN	0.84	0.62 to 1.00	**0.005**	0.77	0.51 to 1.00	0.074	0.54	0.25 to 0.83	0.760	0.56	0.24 to 0.88	0.674	0.84	0.61 to 1.00	**0.021**
PENK	0.94	0.86 to 1.00	**0.0003**	0.92	0.78 to 1.00	**0.005**	0.54	0.28 to 0.79	0.760	0.61	0.32 to 0.90	0.462	0.80	0.55 to 1.00	**0.046**
PPP1R1B	0.66	0.43 to 0.88	0.192	0.78	0.54 to 1.00	0.059	0.59	0.35 to 0.82	0.501	0.78	0.55 to 1.00	0.059	0.81	0.58 to 1.00	**0.036**
SIGMAR1	0.58	0.36 to 0.79	0.514	0.50	0.20 to 0.80	>0.9999	0.59	0.34to 0.85	0.462	0.55	0.25 to 0.84	0.753	0.55	0.25 to 0.85	0.753
TTR	0.60	0.38 to 0.81	0.408	0.67	0.40 to 0.95	0.248	0.59	0.33 to 0.84	0.501	0.72	0.46 to 0.98	0.142	0.72	0.46 to 0.98	0.142
